# Supplementary material for: Bipolar dispersal of red-snow algae
Source: Nat Commun. 2018 Aug 6;9:3094. doi: 10.1038/s41467-018-05521-w (PMC6079020; doi:10.1038/s41467-018-05521-w)
Supplement: Supplementary file 1 — Supplementary information [file 41467_2018_5521_MOESM1_ESM.pdf]

Supplementary Information

Bipolar dispersal of red-snow algae phylotypes

Segawa et al.

This PDF file includes:

Supplementary Figures 1-31

Supplementary Tables 1-12

Supplementary Figures

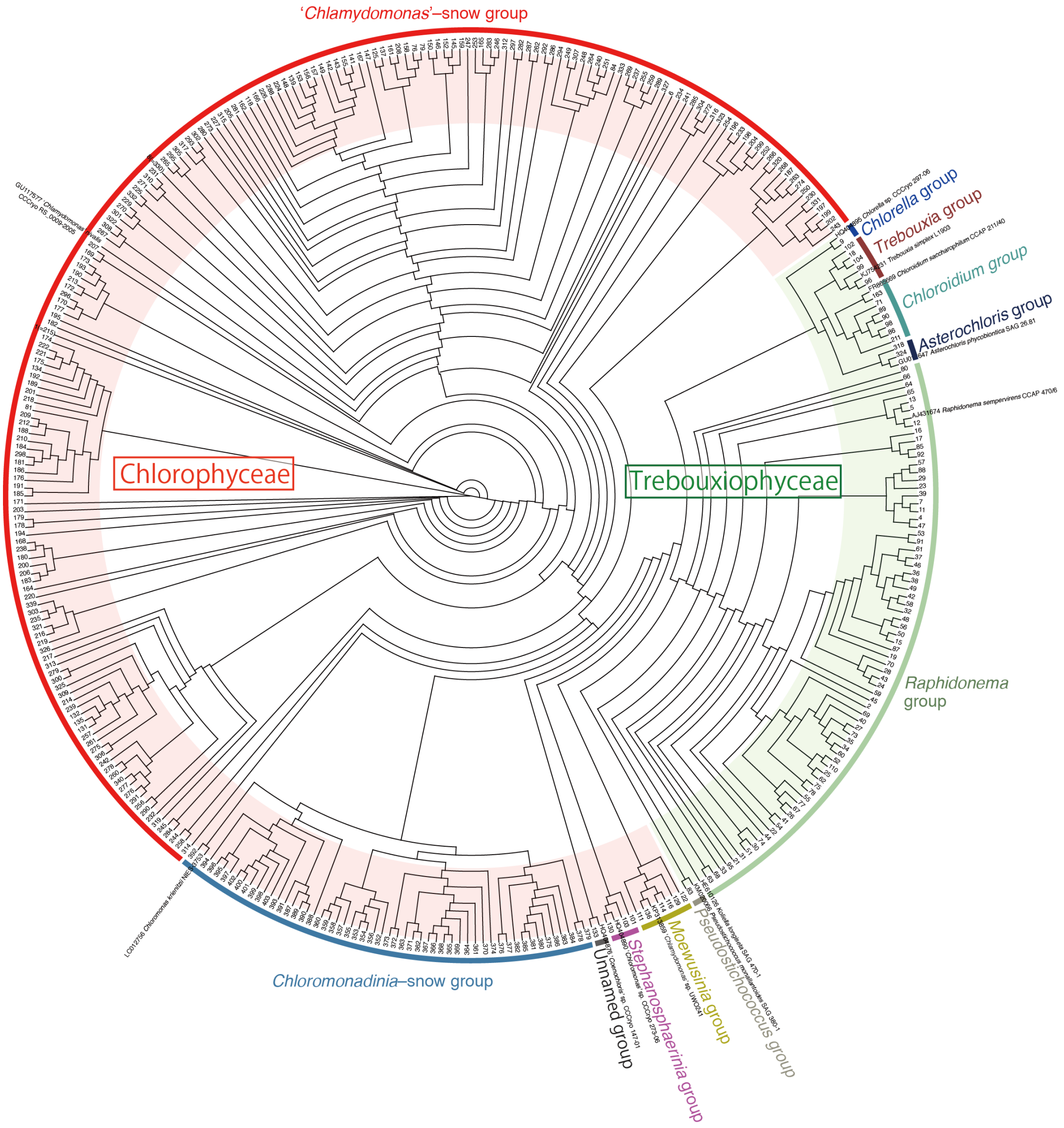

Supplementary Figure 1: Phylogenetic relationship of 348 OTUs (98% OTU) based on ITS2 region sequences. A maximum likelihood tree was constructed with 1000 bootstrap replications using RAxML version 8.2.10 with the GTR+I+Γ model. In 98% OTUs, identical sequences were reduced to a single OTU. Taxonomic groups that were defined by the secondary structure of ITS2 are labeled and distinguished based on colour.

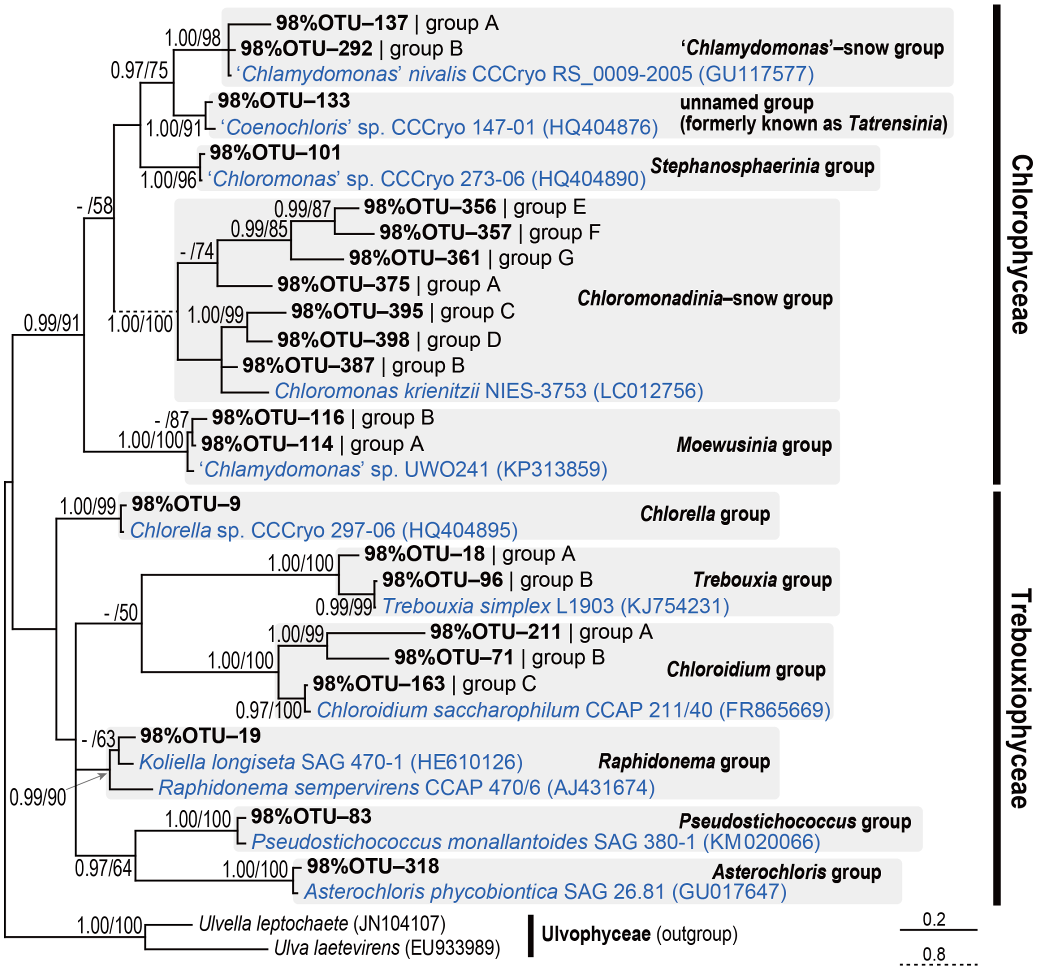

Supplementary Figure 2: Relationship of OTUs within Chlorophyceae or Trebouxiophyceae, based on ITS2 sequences. Bayesian phylogenetic tree was constructed using MrBayes version 3.2.6 with the GTR+I+Γ model. Within each group, species delimitations among the OTUs were carried out on ITS2 secondary structures. The nodal numbers represent posterior probabilities in Bayesian inference (left) and bootstrap values with 1000 replications (right) using RAxML version 8.2.10 with the GTR+I+Γ model. Representative sequences from OTUs generated in this study are shown in bold type.

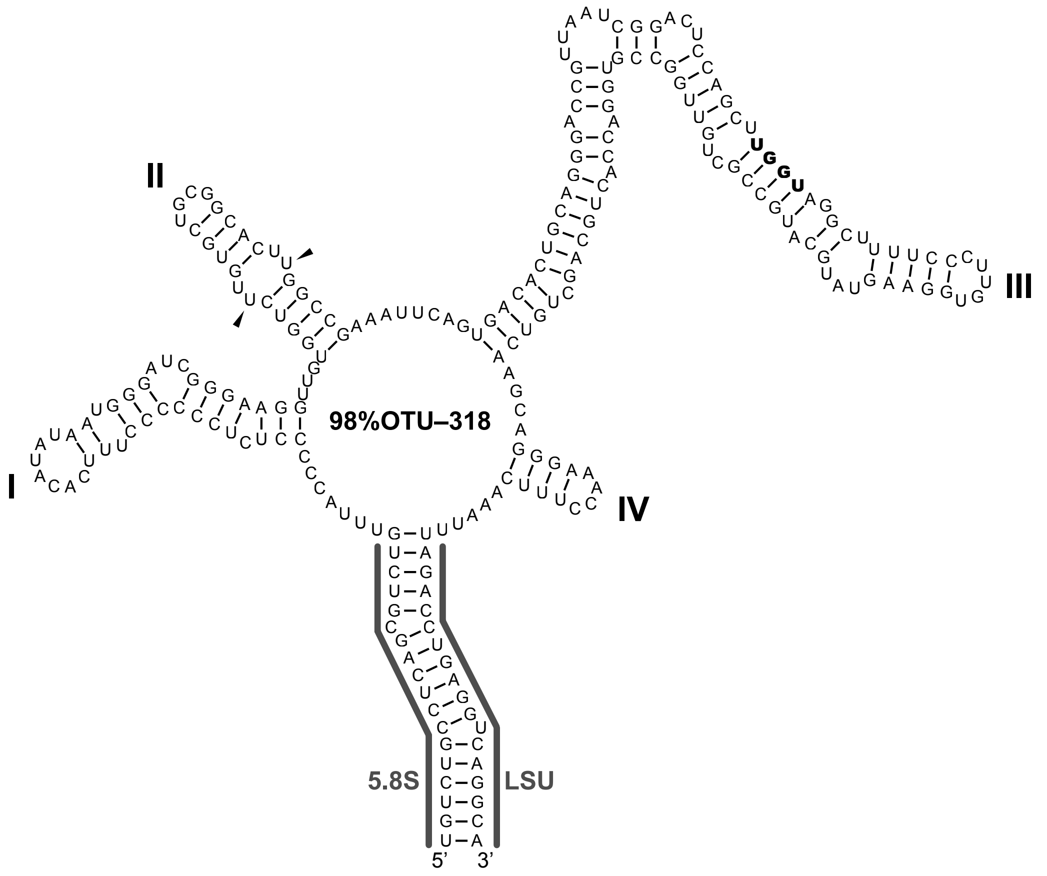

Supplementary Figure 3: Predicted secondary structure of the nuclear rDNA ITS2 transcript of the *Asterochloris* group (98%OTU-318). The 3’ end of the 5.8S ribosomal RNA (rRNA) and the 5’ end of the large subunit of rRNA (LSU rRNA) are indicated by dark lines. Note the U-U mismatch in helix II (arrowheads) and the YGGY motif (UGGU) on the 5’ side of helix III (bold type).

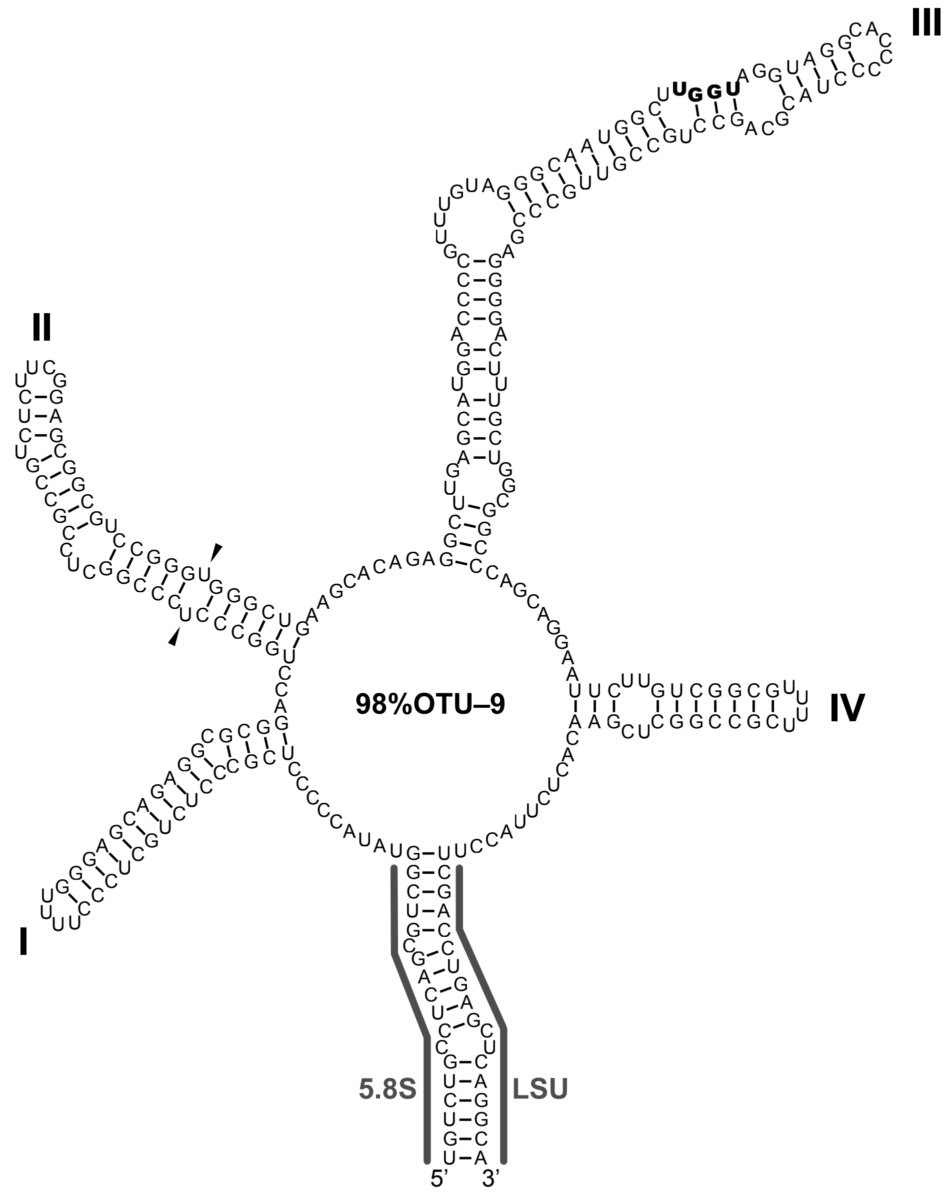

Supplementary Figure 4: Predicted secondary structure of the nuclear rDNA ITS2 transcript of the *Chlorella* group (98%OTU-9). The 3’ end of the 5.8S ribosomal RNA (rRNA) and the 5’ end of the large subunit of rRNA (LSU rRNA) are indicated by dark lines. Note the U-U mismatch in helix II (arrowheads) and the YGGY motif (UGGU) on the 5’ side of helix III (bold type).

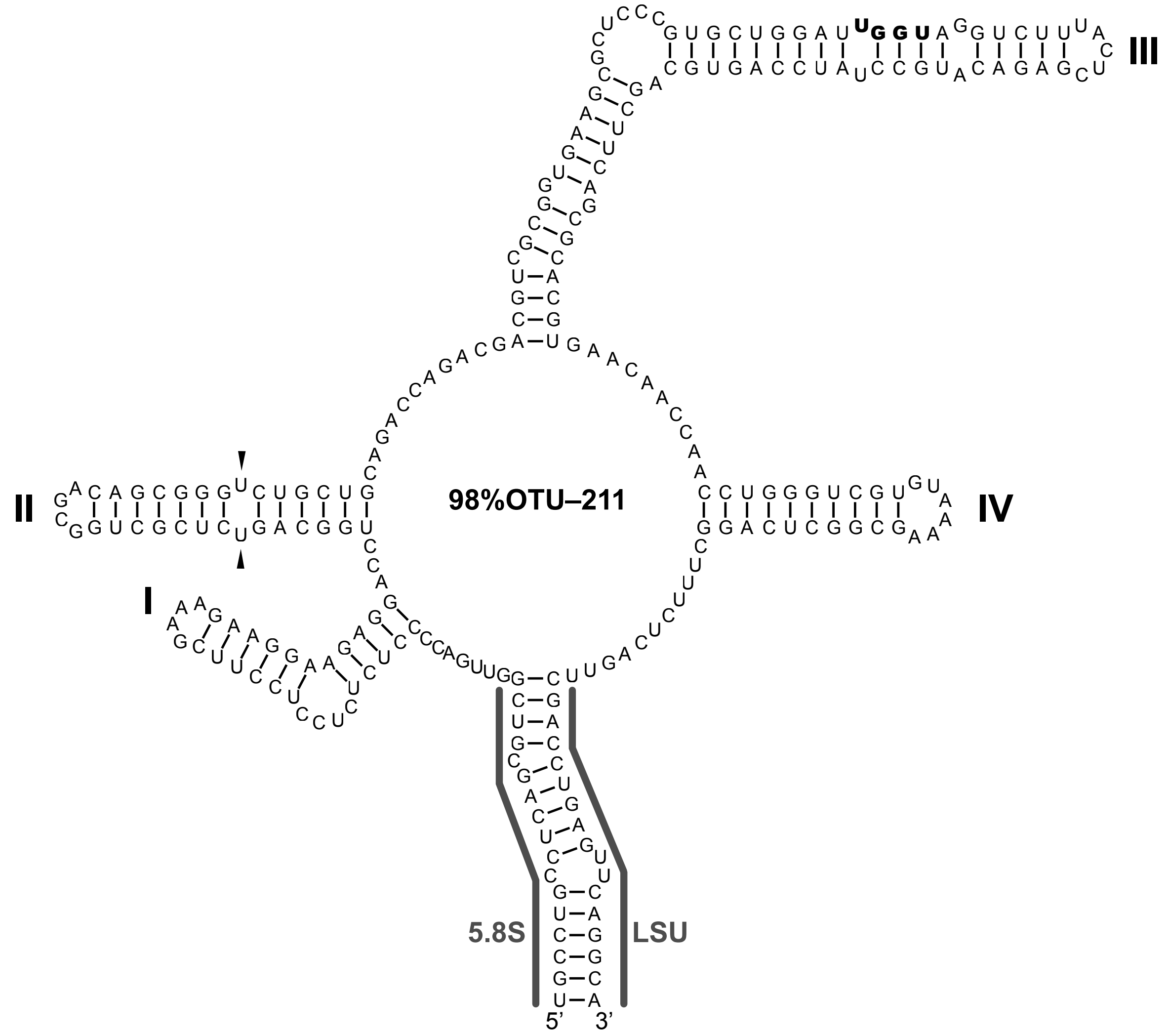

Supplementary Figure 5: Predicted secondary structure of the nuclear rDNA ITS2 transcript of the *Chloroidium* group A (98%OTU-211). The 3’ end of the 5.8S ribosomal RNA (rRNA) and the 5’ end of the large subunit of rRNA (LSU rRNA) are indicated by dark lines. Note the U-U mismatch in helix II (arrowheads) and the YGGY motif (UGGU) on the 5’ side of helix III (bold type).

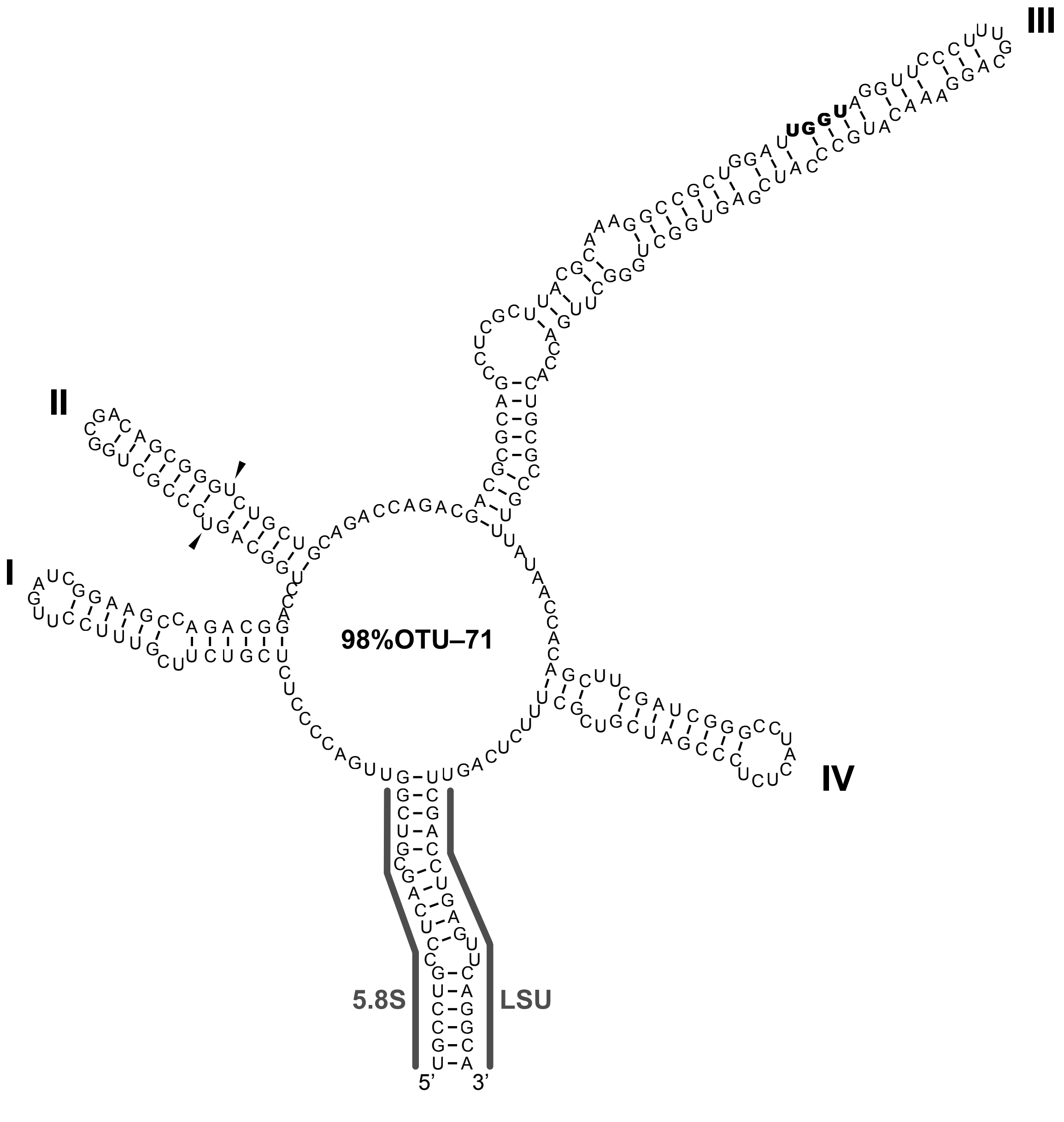

Supplementary Figure 6: Predicted secondary structure of the nuclear rDNA ITS2 transcript of the *Chloroidium* group B (98%OTU-71). The 3’ end of the 5.8S ribosomal RNA (rRNA) and the 5’ end of the large subunit of rRNA (LSU rRNA) are indicated by dark lines. Note the U-U mismatch in helix II (arrowheads) and the YGGY motif (UGGU) on the 5’ side of helix III (bold type).on the 5’ side of helix III (bold type).

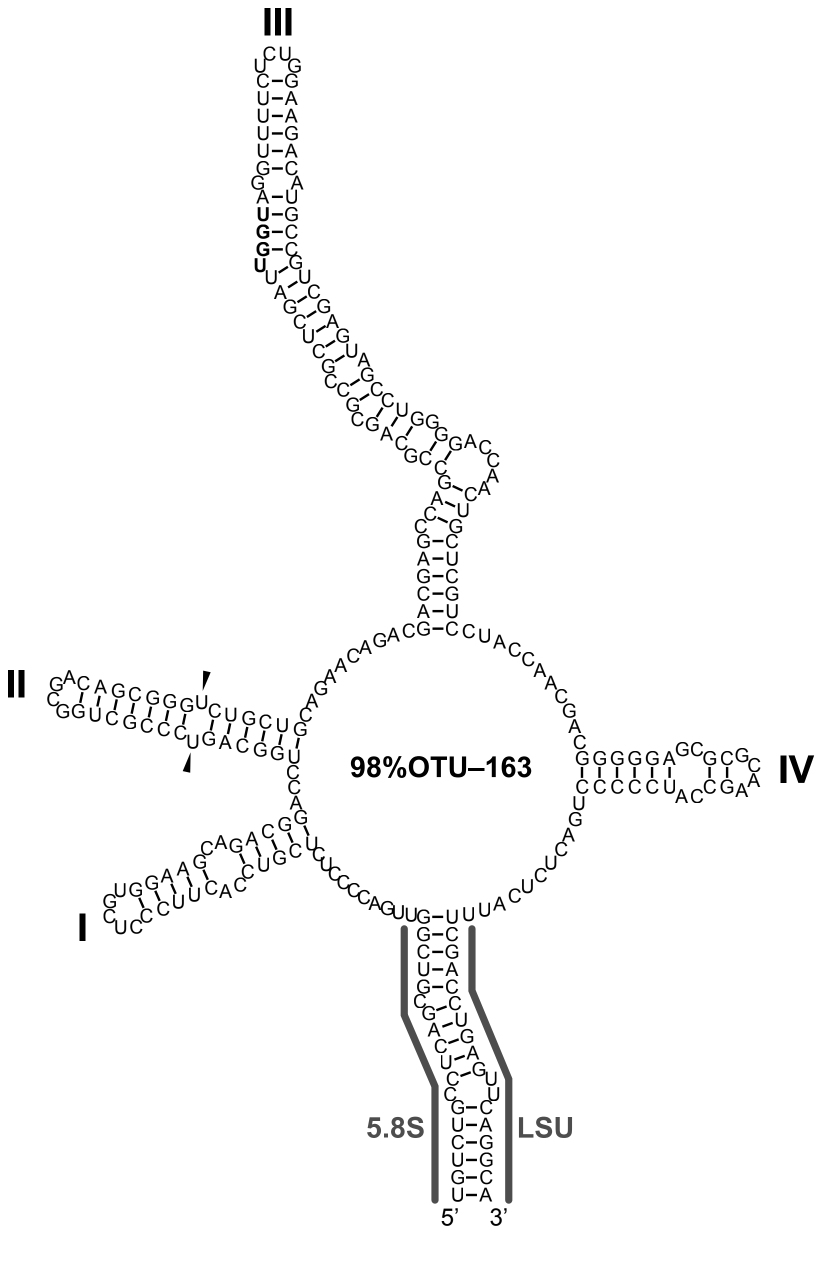

Supplementary Figure 7: Predicted secondary structure of the nuclear rDNA ITS2 transcript of the *Chloroidium* group C (98%OTU-163). The 3’ end of the 5.8S ribosomal RNA (rRNA) and the 5’ end of the large subunit of rRNA (LSU rRNA) are indicated by dark lines. Note the U-U mismatch in helix II (arrowheads) and the YGGY motif (UGGU) on the 5’ side of helix III (bold type).

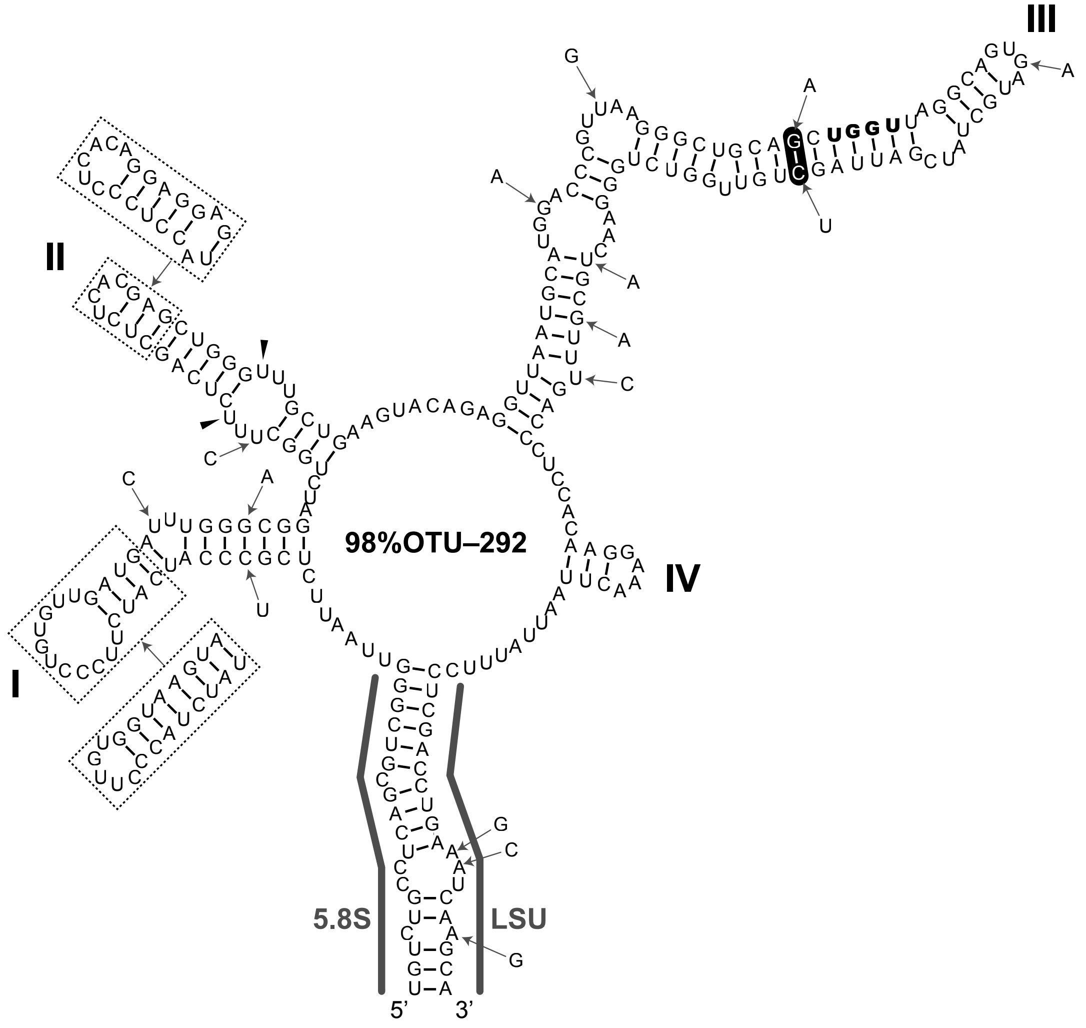

Supplementary Figure 8: Predicted secondary structure of the nuclear rDNA ITS2 transcript of the ‘*Chlamydomonas*’-snow group B (98%OTU-292). Differences between ‘*Chlamydomonas*’-snow group B (98%OTU-292) and ‘*Chlamydomonas*’-snow group A (98%OTU-137) are shown by characters just outside the secondary structure. The 3’ end of the 5.8S ribosomal RNA (rRNA) and the 5’ end of the large subunit of rRNA (LSU rRNA) are indicated by dark lines. The position of the compensatory base change in helix III between the two is shown in a black background. Note the U-U mismatch in helix II (arrowheads) and the YGGY motif (UGGU) on the 5’ side of helix III (bold type).

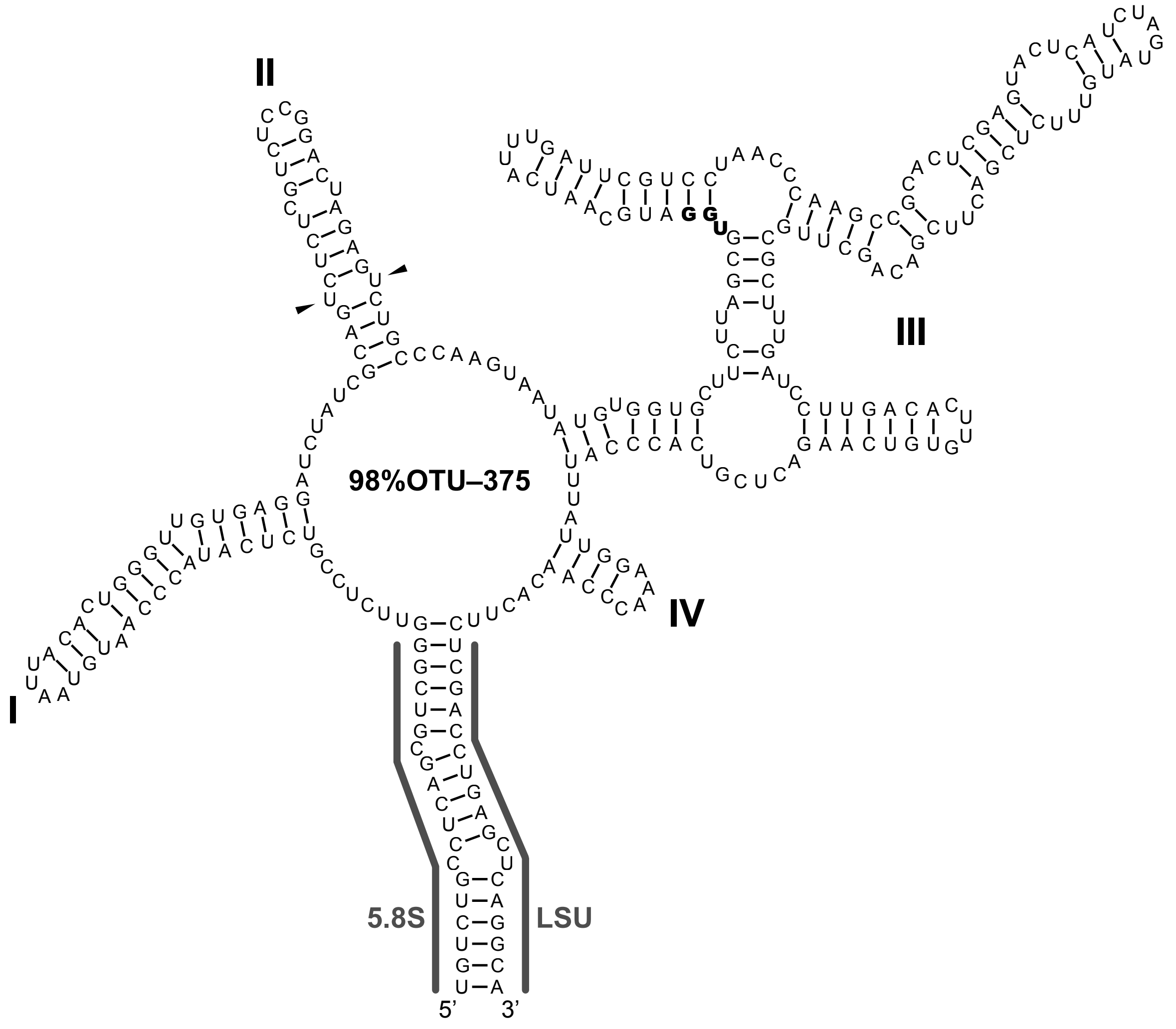

Supplementary Figure 9: Predicted secondary structure of the nuclear rDNA ITS2 transcript of the *Chloromonadinia*-snow group A (98%OTU-375). The 3’ end of the 5.8S ribosomal RNA (rRNA) and the 5’ end of the large subunit of rRNA (LSU rRNA) are indicated by dark lines. Note the U-U mismatch in helix II (arrowheads) and the YGGY motif (UGG) on the 5’ side of helix III (bold type).

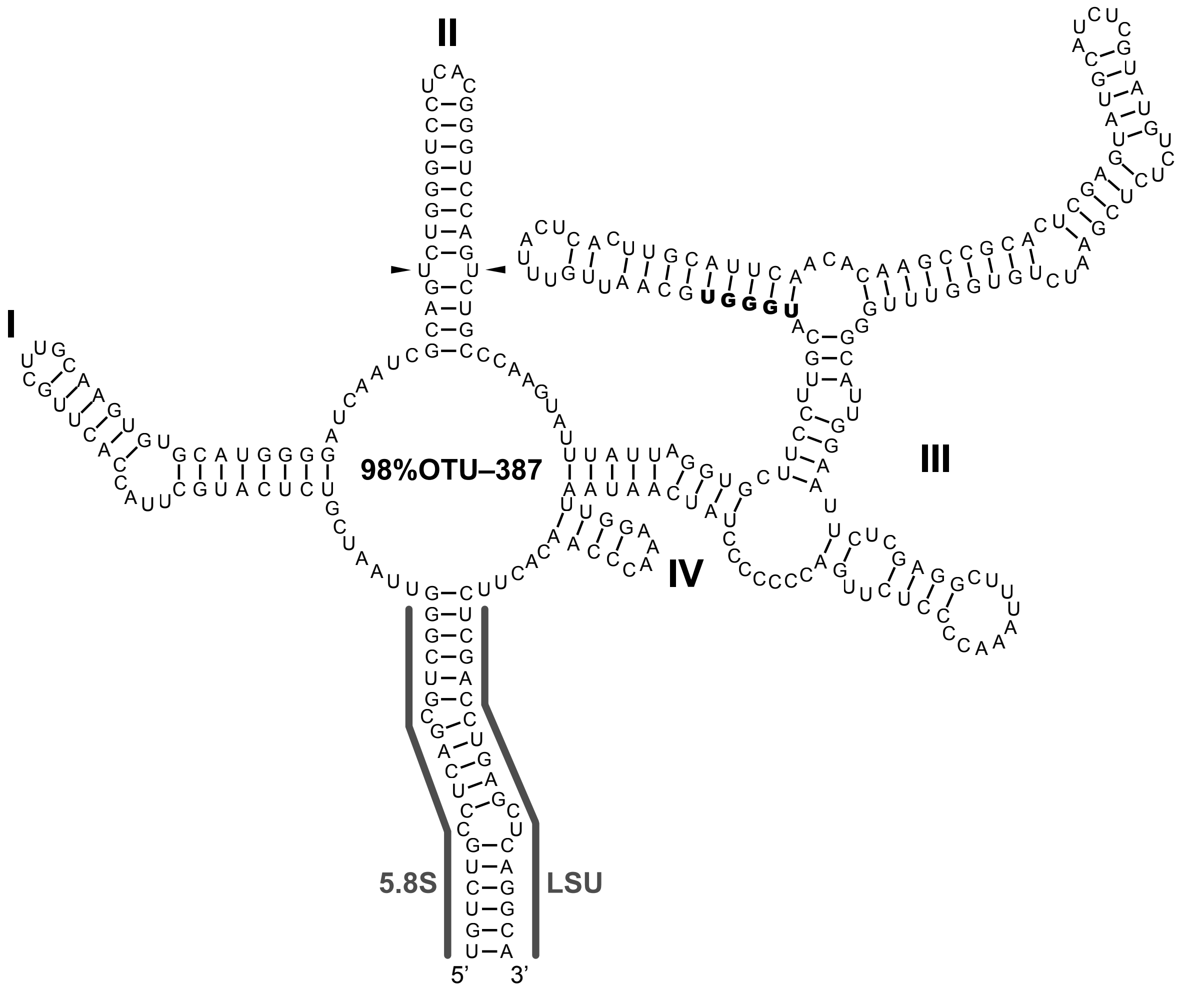

Supplementary Figure 10: Predicted secondary structure of the nuclear rDNA ITS2 transcript of the *Chloromonadinia*-snow group B (98%OTU-387). The 3’ end of the 5.8S ribosomal RNA (rRNA) and the 5’ end of the large subunit of rRNA (LSU rRNA) are indicated by dark lines. Note the U-U mismatch in helix II (arrowheads) and the YGGY motif (UGGGU) on the 5’ side of helix III (bold type).

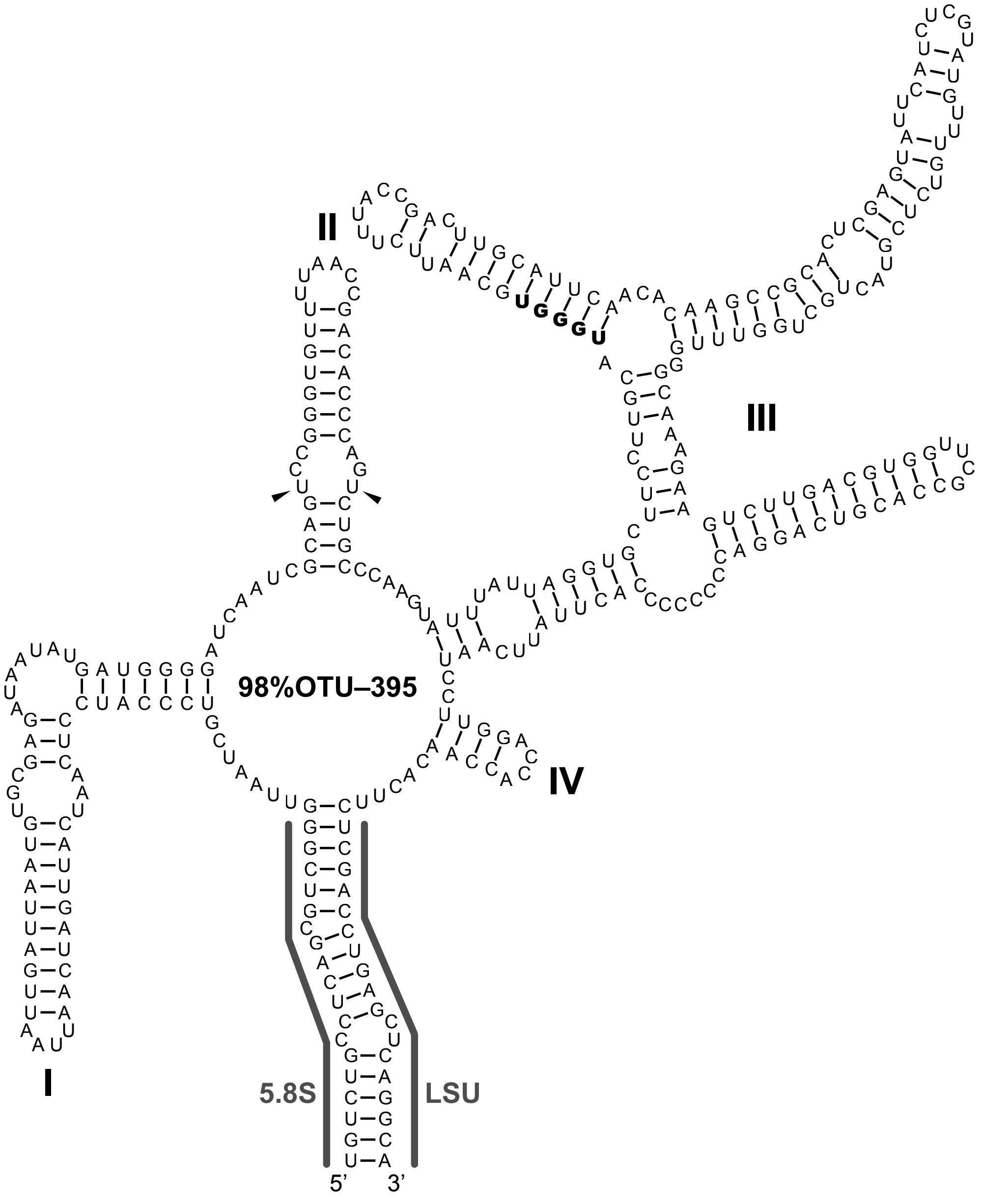

Supplementary Figure 11: Predicted secondary structure of the nuclear rDNA ITS2 transcript of the *Chloromonadinia*-snow group C (98%OTU-395). The 3’ end of the 5.8S ribosomal RNA (rRNA) and the 5’ end of the large subunit of rRNA (LSU rRNA) are indicated by dark lines. Note the U-U mismatch in helix II (arrowheads) and the YGGY motif (UGGGU) on the 5’ side of helix III (bold type).

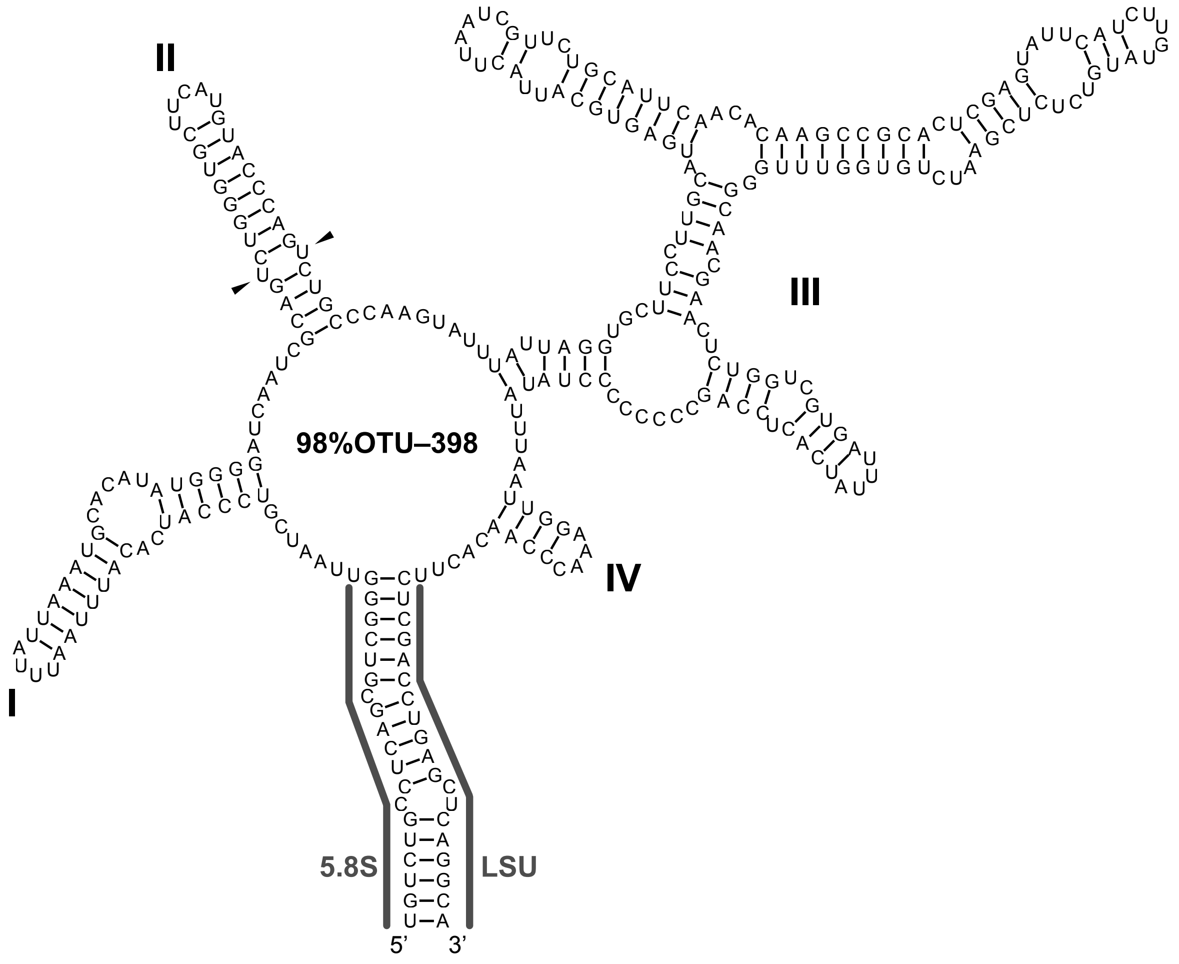

Supplementary Figure 12: Predicted secondary structure of the nuclear rDNA ITS2 transcript of the *Chloromonadinia*-snow group D (98%OTU-398). The 3’ end of the 5.8S ribosomal RNA (rRNA) and the 5’ end of the large subunit of rRNA (LSU rRNA) are indicated by dark lines. Note the U-U mismatch in helix II (arrowheads).

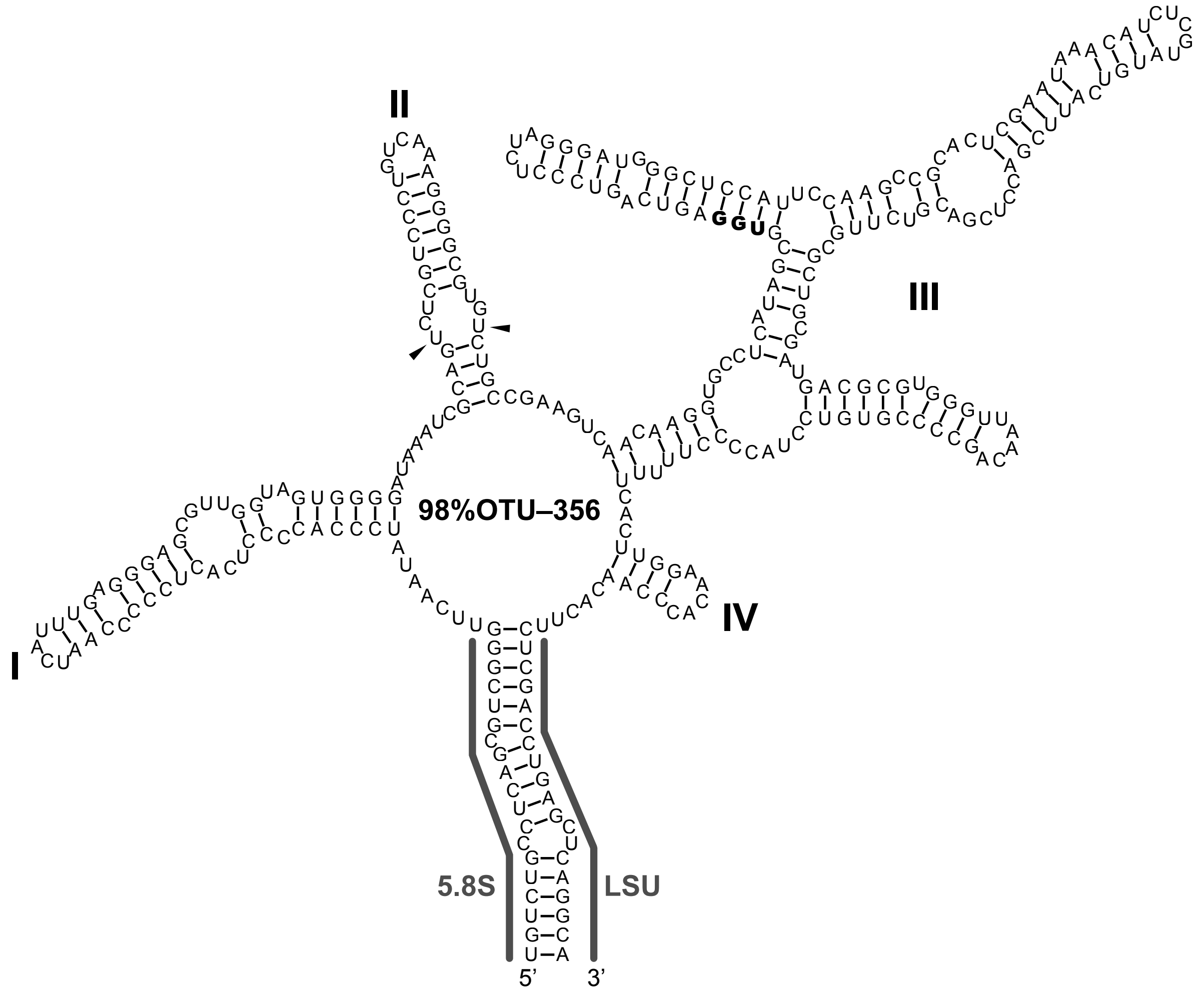

Supplementary Figure 13: Predicted secondary structure of the nuclear rDNA ITS2 transcript of the *Chloromonadinia*-snow group E (98%OTU-356). The 3’ end of the 5.8S ribosomal RNA (rRNA) and the 5’ end of the large subunit of rRNA (LSU rRNA) are indicated by dark lines. Note the U-U mismatch in helix II (arrowheads) and the YGGY motif (UGG) on the 5’ side of helix III (bold type).

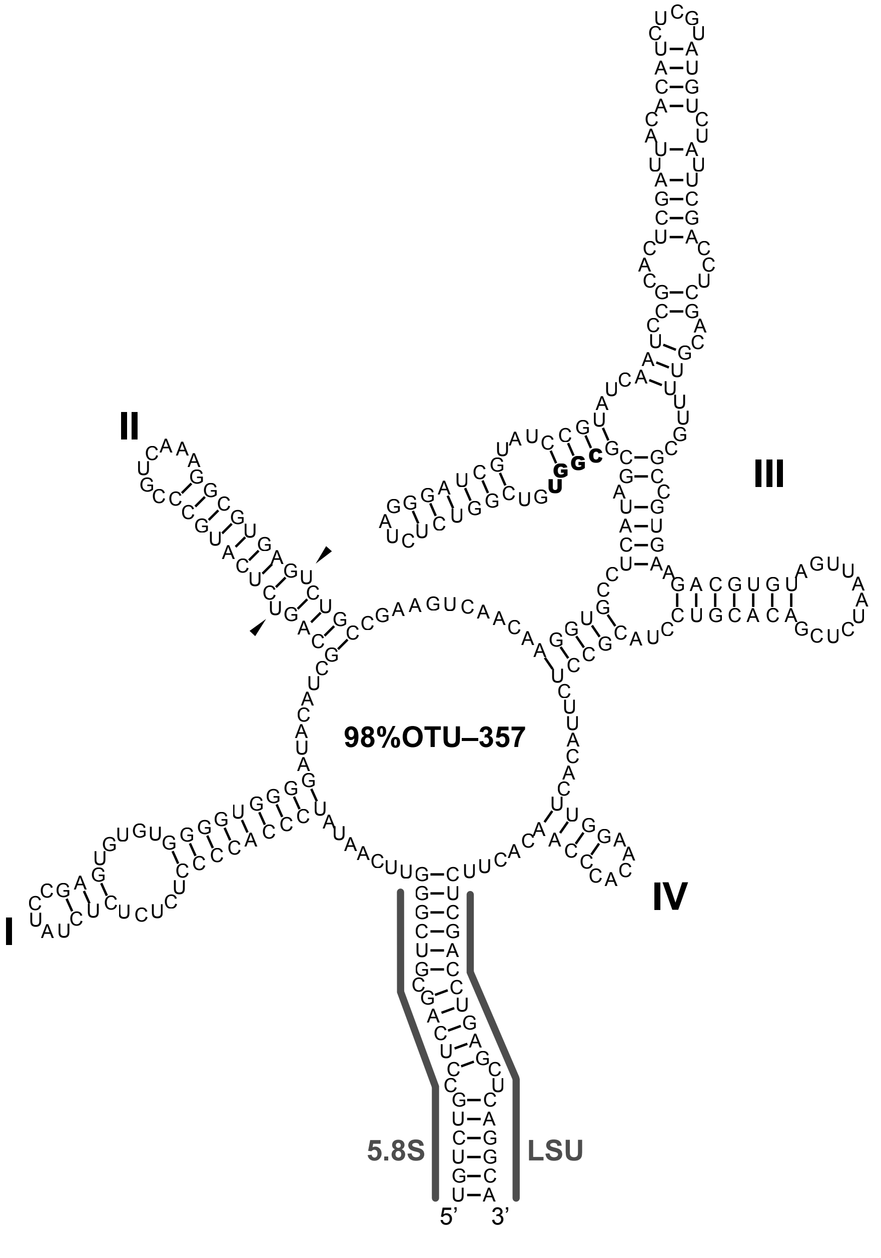

Supplementary Figure 14: Predicted secondary structure of the nuclear rDNA ITS2 transcript of the *Chloromonadinia*-snow group F (98%OTU-357). The 3’ end of the 5.8S ribosomal RNA (rRNA) and the 5’ end of the large subunit of rRNA (LSU rRNA) are indicated by dark lines. Note the U-U mismatch in helix II (arrowheads) and the YGGY motif (CGGU) on the 5’ side of helix III (bold type).

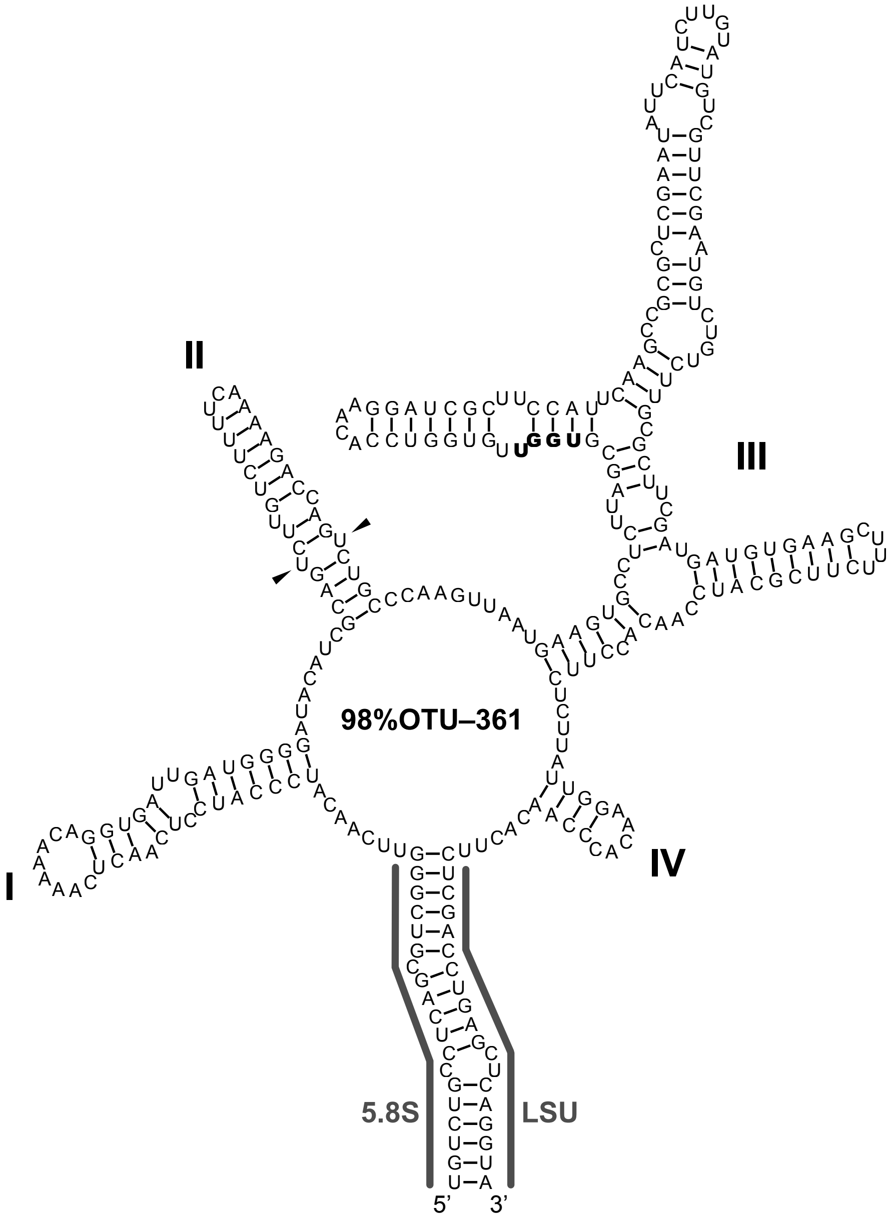

Supplementary Figure 15: Predicted secondary structure of the nuclear rDNA ITS2 transcript of the *Chloromonadinia*-snow group G (98%OTU-361). The 3’ end of the 5.8S ribosomal RNA (rRNA) and the 5’ end of the large subunit of rRNA (LSU rRNA) are indicated by dark lines. Note the U-U mismatch in helix II (arrowheads) and the YGGY motif (UGGU) on the 5’ side of helix III (bold type).

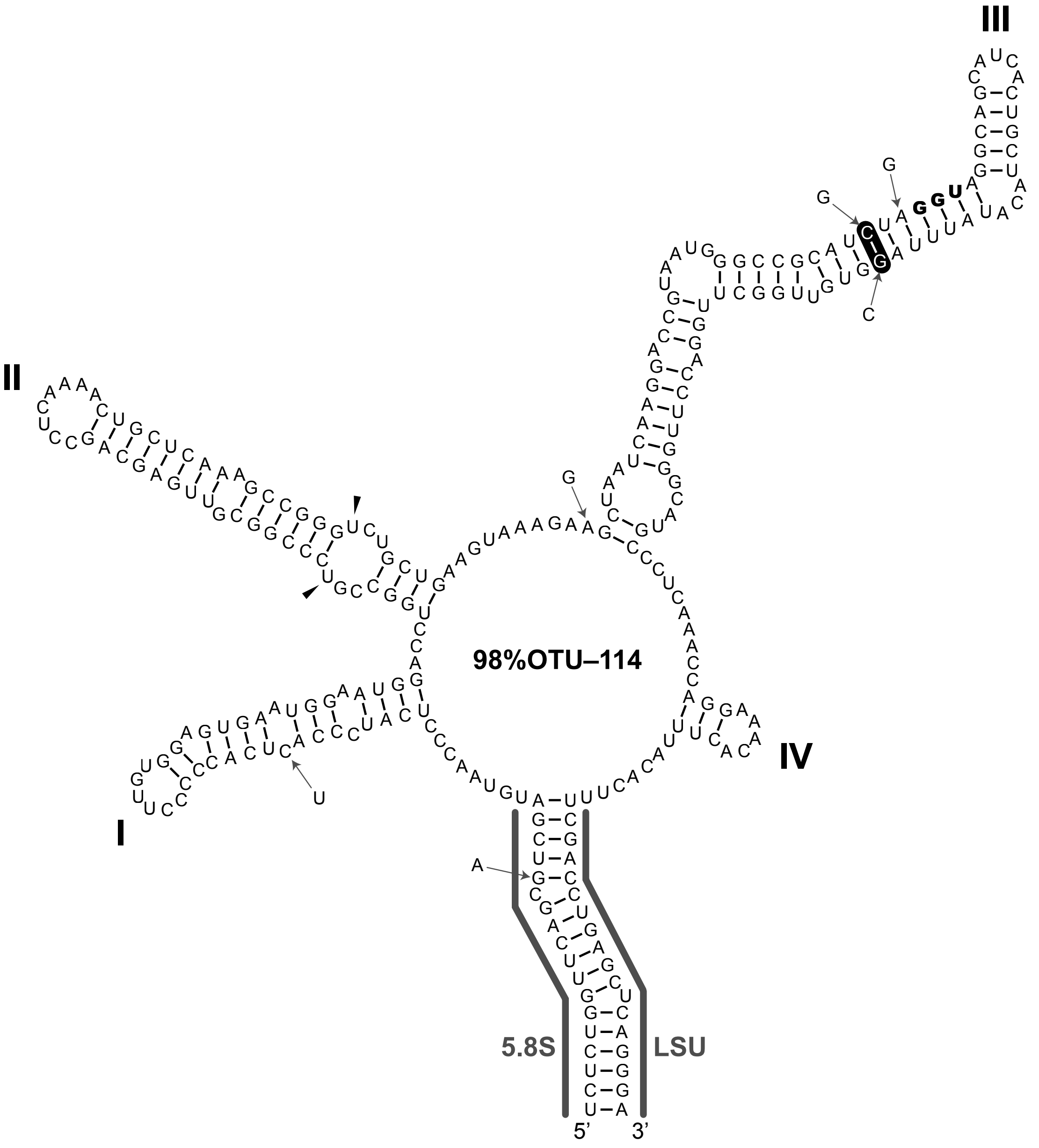

Supplementary Figure 16: Predicted secondary structure of the nuclear rDNA ITS2 transcript of the *Moewusinia* group A (98%OTU-114). Differences between *Moewusinia* group A (98%OTU-114) and *Moewusinia* group B (98%OTU-116) are shown by characters just outside the secondary structure. The 3' end of the 5.8S ribosomal RNA (rRNA) and the 5' end of the large subunit of rRNA (LSU rRNA) are indicated by dark lines. The position of the compensatory base change in helix III between the two is shown in a black background. Note the U-U mismatch in helix II (arrowheads) and the YGGY motif (GGU) on the 5' side of helix III (bold type).

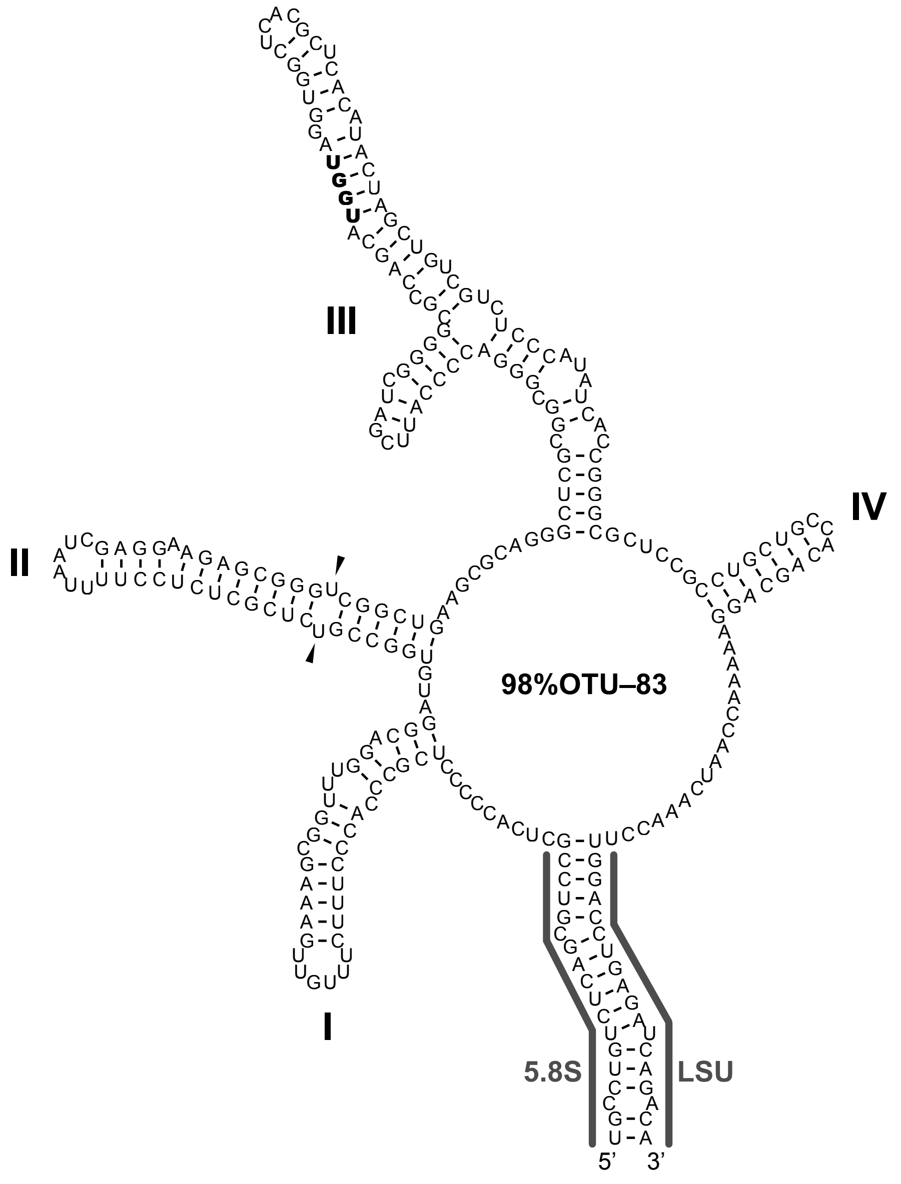

Supplementary Figure 17: Predicted secondary structure of the nuclear rDNA ITS2 transcript of the *Pseudostichococcus* group (98%OTU-83). The 3’ end of the 5.8S ribosomal RNA (rRNA) and the 5’ end of the large subunit of rRNA (LSU rRNA) are indicated by dark lines. Note the U-U mismatch in helix II (arrowheads) and the YGGY motif (UGGU) on the 5’ side of helix III (bold type).

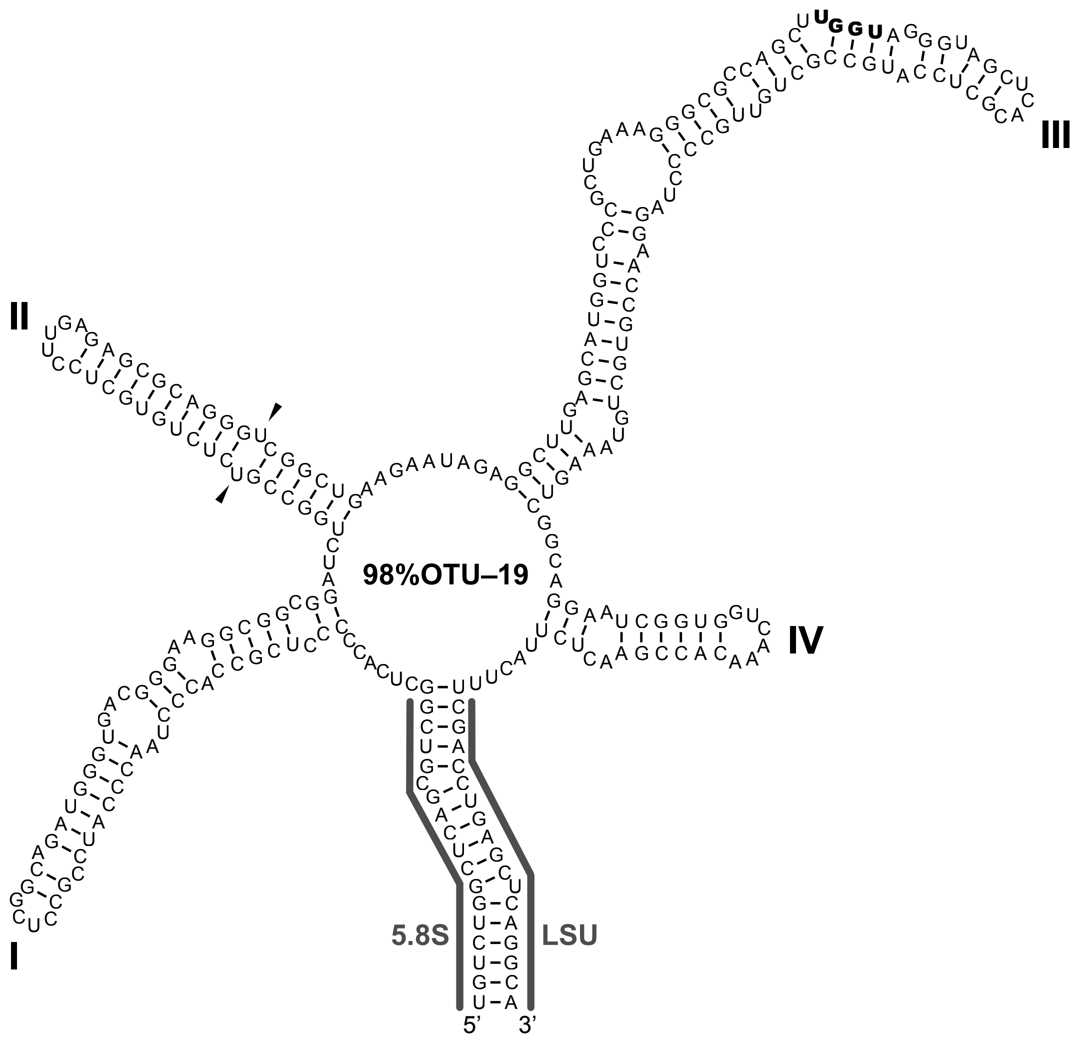

Supplementary Figure 18: Predicted secondary structure of the nuclear rDNA ITS2 transcript of the *Raphidonema* group (98%OTU-19). The 3’ end of the 5.8S ribosomal RNA (rRNA) and the 5’ end of the large subunit of rRNA (LSU rRNA) are indicated by dark lines. Note the U-U mismatch in helix II (arrowheads) and the YGGY motif (UGGU) on the 5’ side of helix III (bold type).

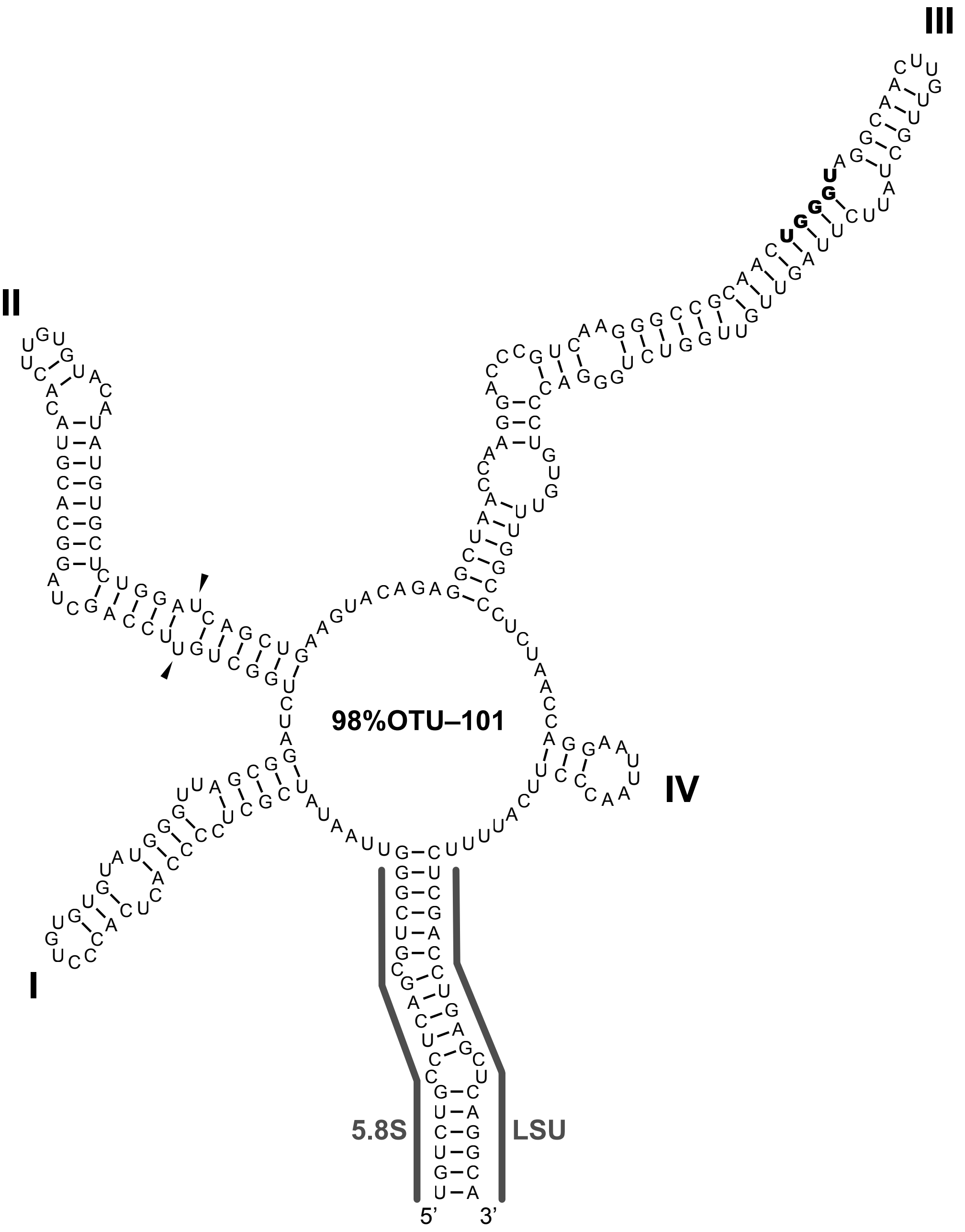

Supplementary Figure 19: Predicted secondary structure of the nuclear rDNA ITS2 transcript of the *Stephanosphaerinia* group (98%OTU-101). The 3’ end of the 5.8S ribosomal RNA (rRNA) and the 5’ end of the large subunit of rRNA (LSU rRNA) are indicated by dark lines. Note the U-U mismatch in helix II (arrowheads) and the YGGY motif (UGGGU) on the 5’ side of helix III (bold type).

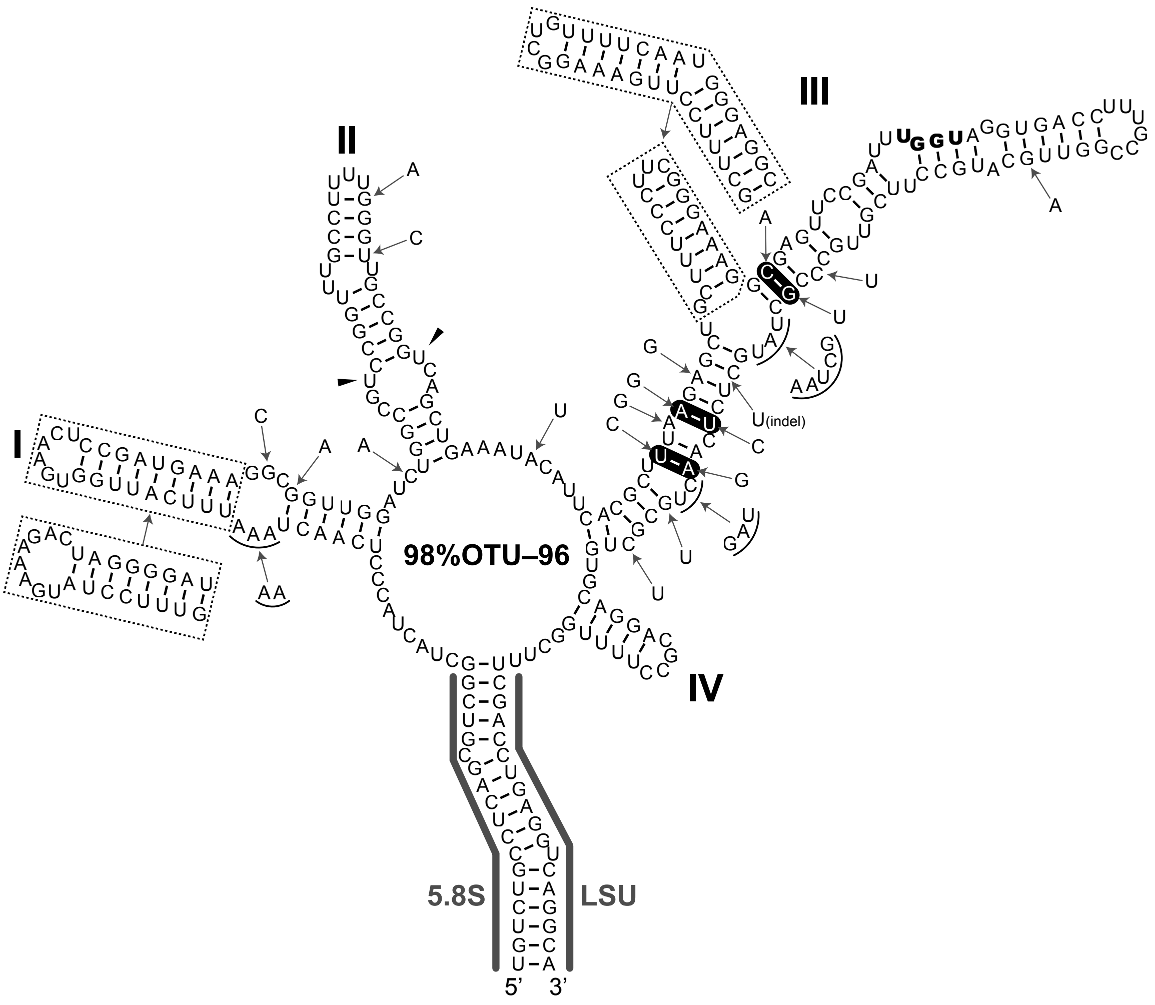

Supplementary Figure 20: Predicted secondary structure of the nuclear rDNA ITS2 transcript of the *Trebouxia* group B (98%OTU-96). Differences between *Trebouxia* group B (98%OTU-96) and *Trebouxia* group A (98%OTU-18) are shown by characters just outside the secondary structure. The 3’ end of the 5.8S ribosomal RNA (rRNA) and the 5’ end of the large subunit of rRNA (LSU rRNA) are indicated by dark lines. The positions of the compensatory base changes in helix III between the two are shown in black backgrounds. Note the U-U mismatch in helix II (arrowheads) and the YGGY motif (UGGU) on the 5’ side of helix III (bold type).

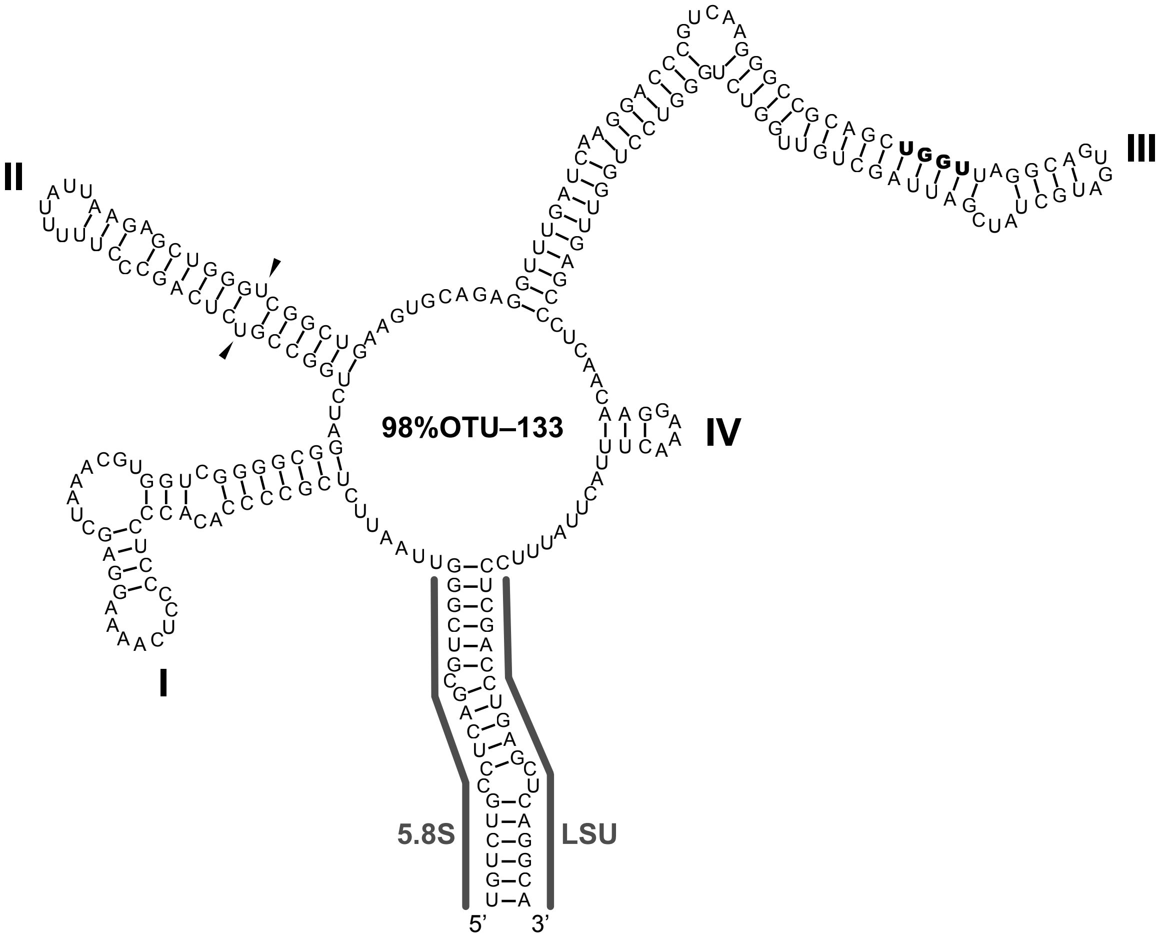

Supplementary Figure 21: Predicted secondary structure of the nuclear rDNA ITS2 transcript of the unnamed group (98%OTU-133). The 3’ end of the 5.8S ribosomal RNA (rRNA) and the 5’ end of the large subunit of rRNA (LSU rRNA) are indicated by dark lines. Note the U-U mismatch in helix II (arrowheads) and the YGGY motif (UGGU) on the 5’ side of helix III (bold type).

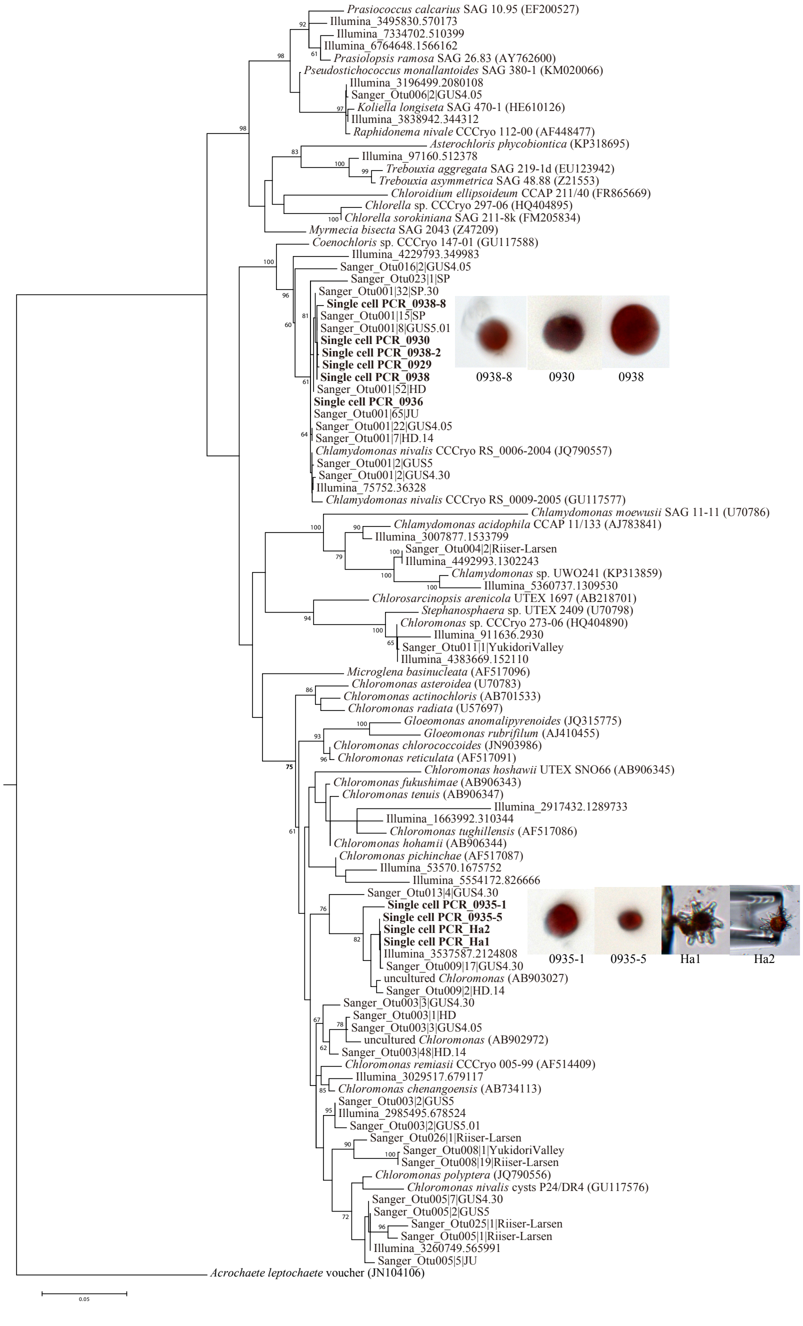

Supplementary Figure 22: Phylogenetic relationship between the single-cell, Sanger clone and Illumina sequences of 18S rRNA gene. A maximum likelihood tree was constructed based on the 18S rRNA gene sequences with 1000 bootstrap replications using RAxML version 8.2.10 with the GTR+ I+Γ model. *Acrochaete leptochaete* was used as an outgroup. Sequences from single-cell PCR obtained in this study are shown in bold type.

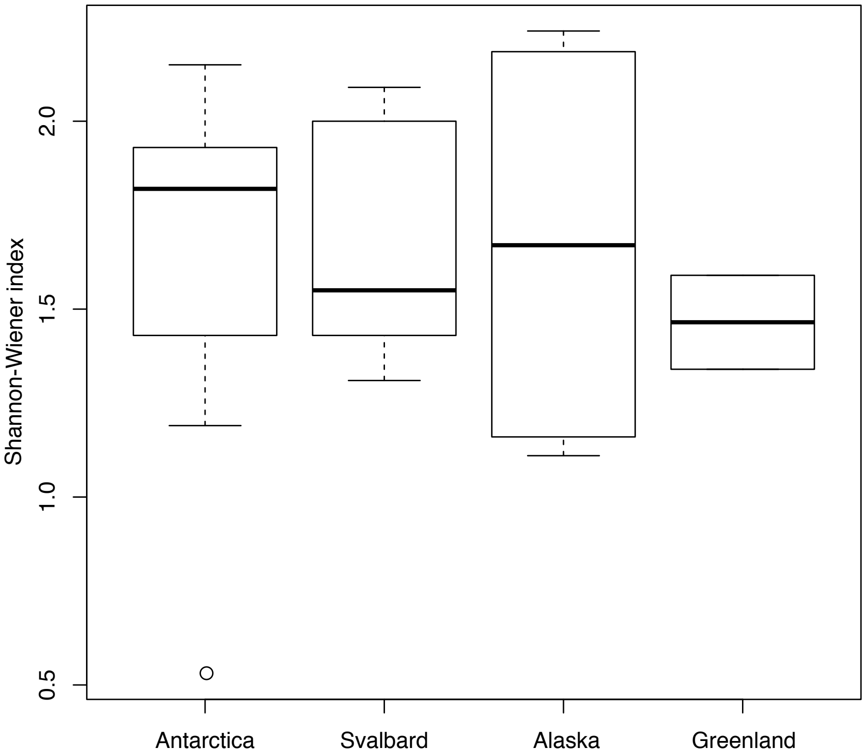

Supplementary Figure 23: Shannon-Wiener index of snow algae communities in the various regions based on 98% OTU. The box plot indicates the median, first quantile, third quantile, and outliers. There were no significant differences between regions ($P > 0.05$; Student's t-test).

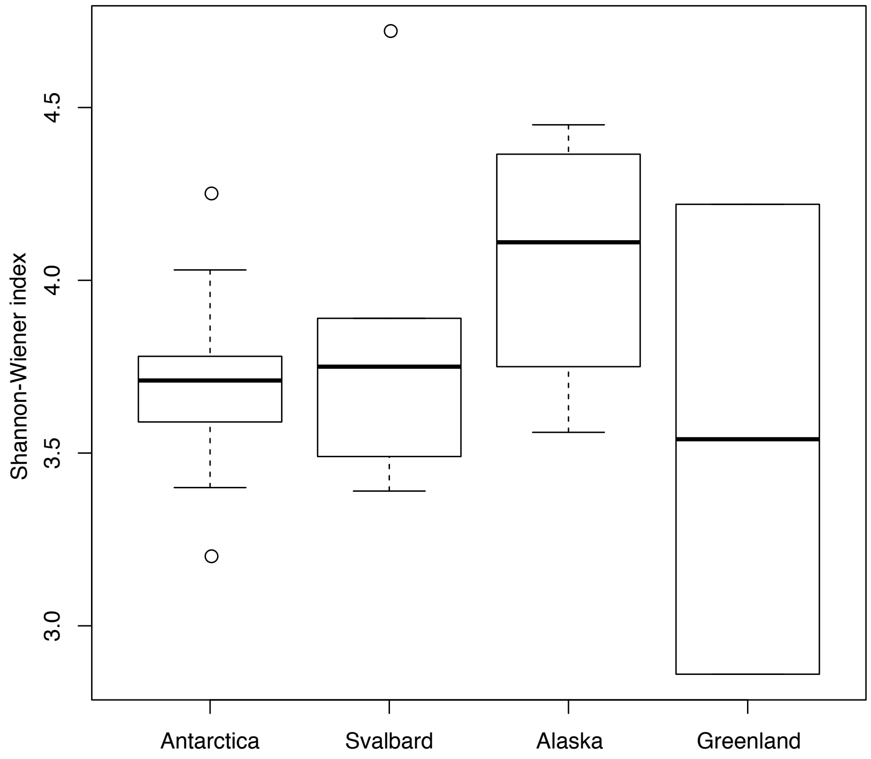

Supplementary Figure 24: Shannon-Wiener index of snow algae communities in the various regions based on unique sequences. The box plot indicates the median, first quantile, third quantile, and outliers. There were no significant differences between regions ($P > 0.05$; Student's t-test).

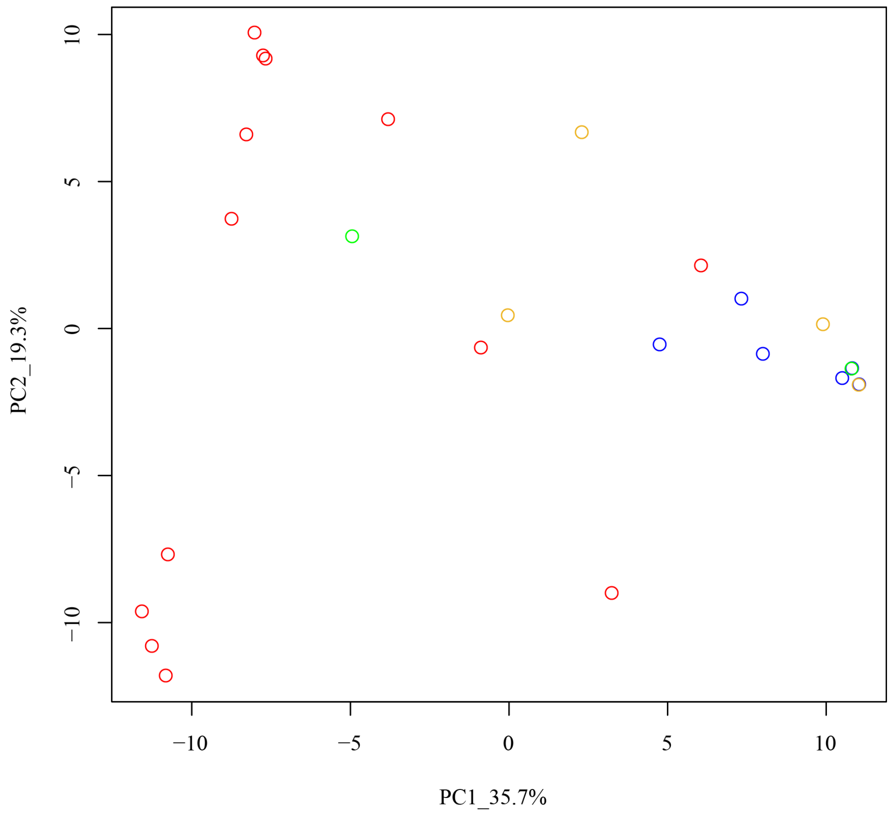

Supplementary Figure 25: Similarities among the ITS2 regions of unique sequences were evaluated by principal components analysis (PCA). Different algal community structure between samples as shown by PCA plot. The PCA plot was generated using the abundance matrix of the unique ITS2 sequences obtained from red snow samples. Each coloured point represents an amplicon from the red snow samples obtained from the following regions. red: Antarctica, green: Greenland, blue: Svalbard, yellow: Alaska.

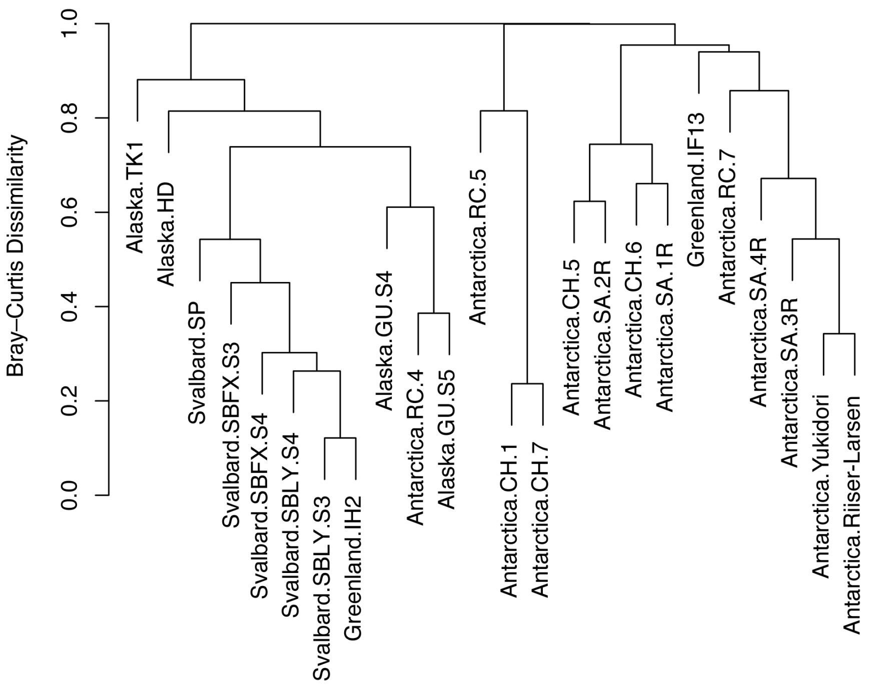

Supplementary Figure 26: Bray-Curtis dissimilarity based on 98% OTU. The clustering figure shows clear separation of algal communities between the Arctic and Antarctica, indicating that geographical distribution has been affected by geographical distance.

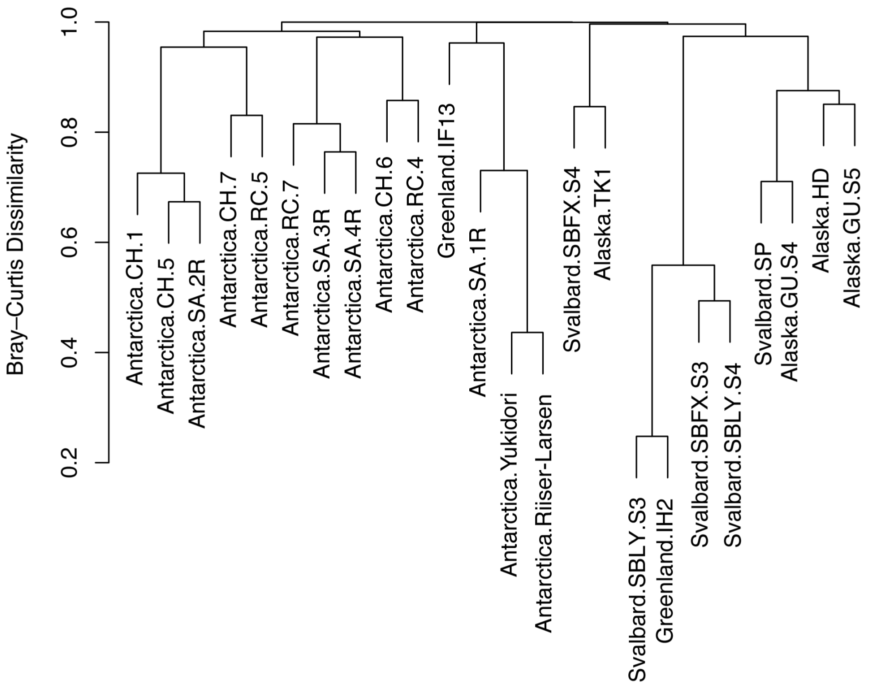

Supplementary Figure 27: Bray-Curtis dissimilarity based on unique sequences. The clustering figure shows clear separation of algal communities between the Arctic and Antarctica, indicating that geographical distribution has been affected by geographical distance.

.

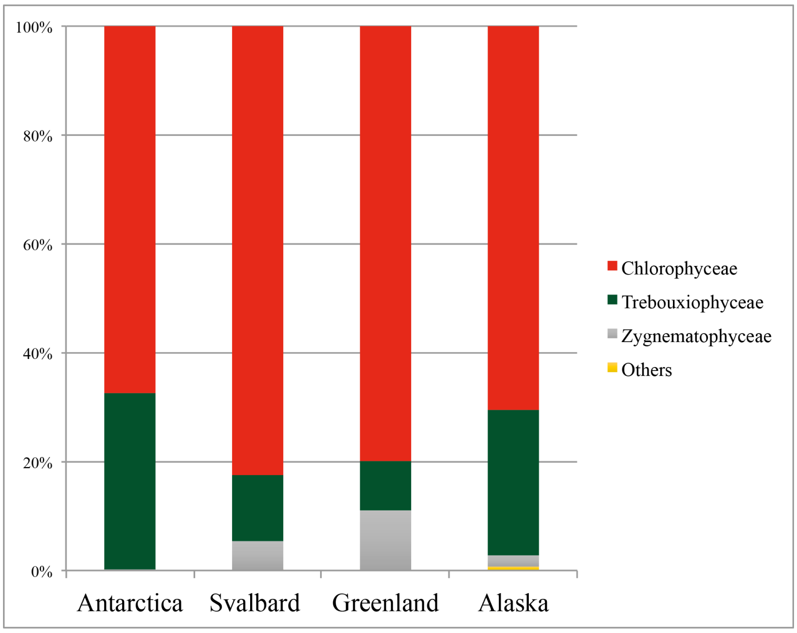

Supplementary Figure 28: Algal taxonomic compositions in the red snows based on 18S rRNA gene by Illumina sequences.

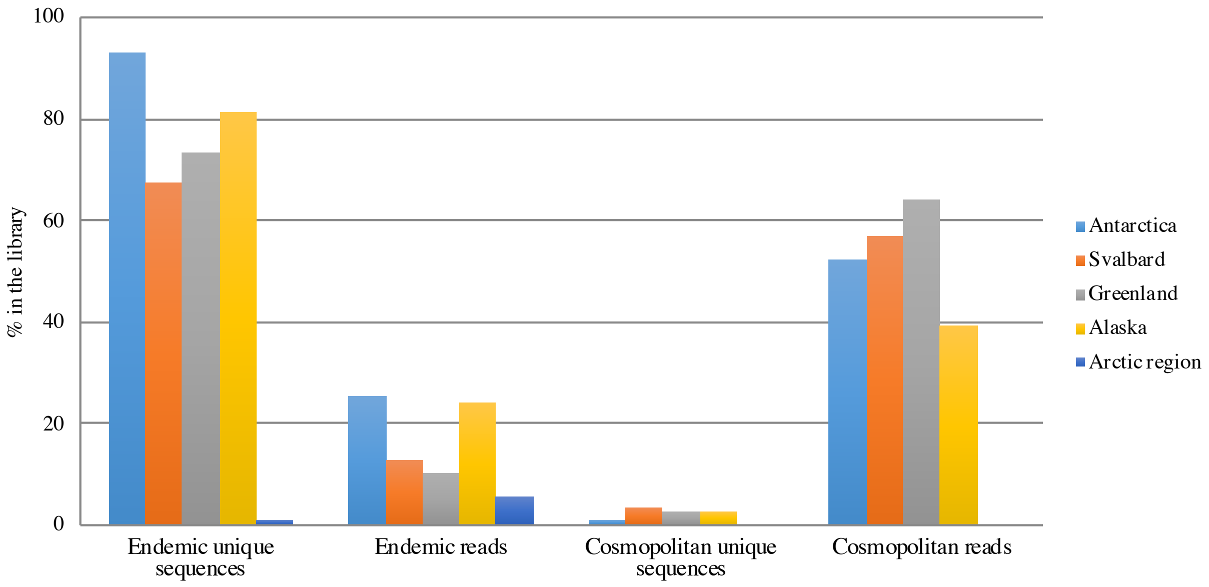

Supplementary Figure 29: Distribution pattern for endemic and cosmopolitan snow algae based on 18S rRNA gene (unique sequences) obtained from each region. Unique sequence numbers and read numbers from Illumina sequences are shown.

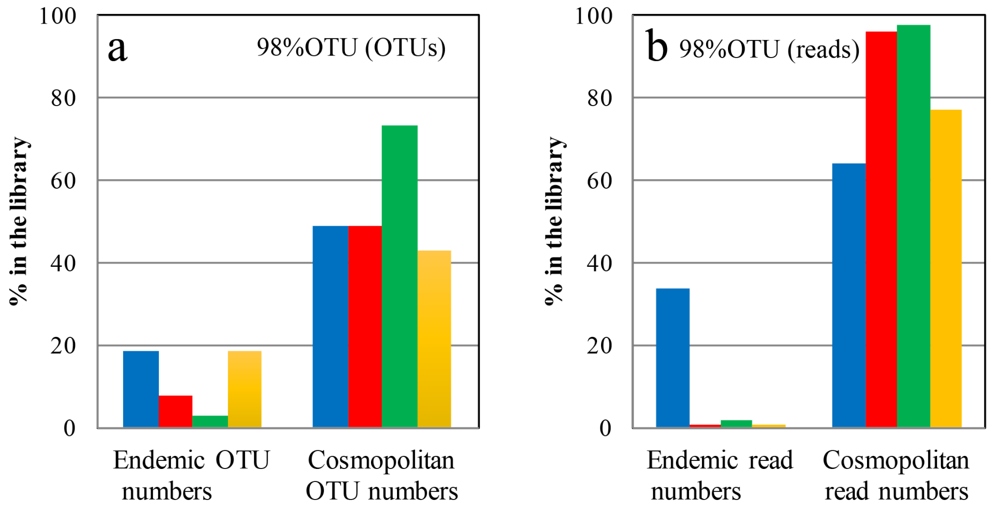

Supplementary Figure 30: Distribution pattern for the endemic and bipolar cosmopolitan snow algae based on ITS2 sequences (98% OTU) from Illumina sequencing obtained from each region. OTUs and sequencing read numbers are shown. a, Number of OTUs. b, read numbers of OTUs. Bars are coloured according to region in which the snow sample was obtained from a glacier and snow patch: blue: Antarctica, red: Svalbard, green: Greenland, and yellow: Alaska.

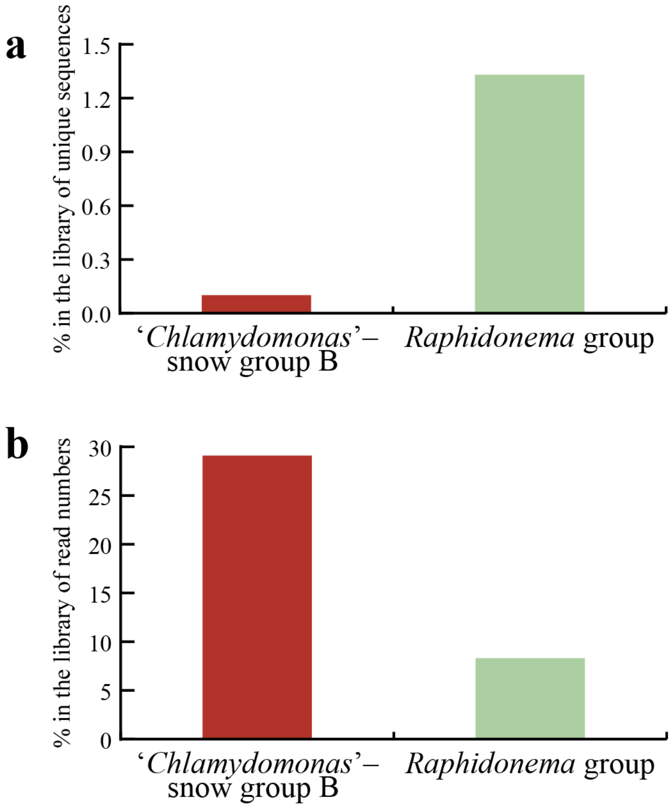

Supplementary Figure 31: Bipolar distribution in '*Chlamydomonas*'-snow group B and *Raphidonema* group of snow algal unique sequences. a, Unique sequences in total. b, Sequencing read numbers in total.

Supplementary Tables

Supplementary Table 1: List of red snow samples used in this study

Region	Sample location	Site	Lat	Long	Altitude (m)	Year	Site condition
Greenland	Greenland Ice sheet, Tugto Glacier	IF13	77.89473	-68.77335	1001	2012.July	snow on the glacier
Greenland	Greenland Ice sheet	IH2	69.56110	-49.34715	1107	2011.August	snow on the glacier
Svalbard	Foxfonna glacier	SBFX.S3	78.13747	16.15794	642	2011.August	snow on the glacier
Svalbard	Foxfonna glacier	SBFX.S4	78.12687	16.20279	755	2011.August	snow on the glacier
Svalbard	Longyearbreen glacier	SBLY.S3	78.17579	15.49680	417	2011.August	snow on the glacier
Svalbard	Longyearbreen glacier	SBLY.S4	78.16565	15.46344	580	2011.August	snow on the glacier
Svalbard	Austre Brøggerbreen	SP	78.89904	11.82895	130	2008.July	snow on the glacier
Alaska	Juneau ice field	TK1	58.64446	-134.21068	1040	2001.July	snow on the glacier
Alaska	Gulkana glacier	GU.S5	63.28771	-145.40551	1680	2001.August	snow on the glacier
Alaska	Gulkana glacier	GU.S4	63.28068	-145.41242	1585	2005.August	snow on the glacier
Alaska	Harding ice field	HD	60.15278	-149.77992	1150	2014.August	snow on the glacier
Antarctica	Riiser-Larsen	Riiser-Larsen	-66.78765	50.56543	500	2008.February	non-glacier based seasonal snow
Antarctica	Yukidori Valley	Yukidori	-69.23935	39.76112	50	2008.January	non-glacier based seasonal snow
Antarctica	Johnsons Glacier, Livingston Island	CH.1	-62.65595	-60.36640	5	2015.January	non-glacier based seasonal snow
Antarctica	Johnsons Glacier, Livingston Island	CH.5	-62.65588	-60.36582	5	2015.January	non-glacier based seasonal snow
Antarctica	Johnsons Glacier, Livingston Island	CH.6	-62.65563	-60.36594	5	2015.January	non-glacier based seasonal snow
Antarctica	Johnsons Glacier, Livingston Island	CH.7	-62.65604	-60.36558	5	2015.January	non-glacier based seasonal snow
Antarctica	Glaciar Rocoso, Livingston Island	RC.4	-62.71219	-60.40879	35	2015.January	non-glacier based seasonal snow
Antarctica	Glaciar Rocoso, Livingston Island	RC.5	-62.71250	-60.40926	30	2015.January	non-glacier based seasonal snow
Antarctica	Glaciar Rocoso, Livingston Island	RC.7	-62.71508	-60.41130	10	2015.January	non-glacier based seasonal snow
Antarctica	Hurd Glacier-Sally Rocks lobe, Livingston Island	SA.1R	-62.70138	-60.41831	35	2015.January	non-glacier based seasonal snow
Antarctica	Hurd Glacier-Sally Rocks lobe, Livingston Island	SA.2R	-62.70153	-60.41784	12	2015.January	non-glacier based seasonal snow
Antarctica	Hurd Glacier-Sally Rocks lobe, Livingston Island	SA.3R	-62.70243	-60.41806	0	2015.January	non-glacier based seasonal snow
Antarctica	Hurd Glacier-Sally Rocks lobe, Livingston Island	SA.4R	-62.70299	-60.41846	0	2015.January	non-glacier based seasonal snow

Supplementary Table 2: Numbers and ratio for each group based on Illumina unique sequences and 98% OTU analysis, respectively

	98% OTU numbers	98% OTU (%)	Unique sequences numbers	Unique sequences (%)
'*Chlamydomonas*'-snow group A	33	9.5	5946	9.3
'*Chlamydomonas*'-snow group B	163	46.8	36701	57.3
unnamed group	1	0.3	1	0.0
Stephanosphaerinia group	3	0.9	49	0.1
Moewusinia group A	5	1.4	137	0.2
Moewusinia group B	1	0.3	1	0.0
Chlorella group	1	0.3	5	0.0
Raphidonema group	74	21.3	15515	24.2
Trebouxia group A	3	0.9	5	0.0
Trebouxia group B	2	0.6	16	0.0
Chloroidium group A	1	0.3	2	0.0
Chloroidium group B	5	1.4	14	0.0
Chloroidium group C	1	0.3	1	0.0
Pseudostichococcus group	1	0.3	1	0.0
Asterochloris group	2	0.6	9	0.0
Chloromonadinia-snow group A	12	3.4	418	0.7
Chloromonadinia-snow group B	8	2.3	387	0.6
Chloromonadinia-snow group C	3	0.9	247	0.4
Chloromonadinia-snow group D	6	1.7	24	0.0
Chloromonadinia-snow group E	5	1.4	39	0.1
Chloromonadinia-snow group F	4	1.1	140	0.2
Chloromonadinia-snow group G	14	4.0	4389	6.9

Supplementary Table 3: Number of Illumina raw reads for each taxonomy group in the various regions

	Antarctica	Svalbard	Greenland	Alaska	Total
'*Chlamydomonas*'-snow group A	136,038	36,754	5,291	4,332	182,415
'*Chlamydomonas*'-snow group B	156,173	1,012,397	221,624	687,329	2,077,523
unnamed group	16	0	0	0	16
Stephanosphaerinia group	386	0	0	0	386
Moewusinia group A	1,598	47	0	0	1,645
Moewusinia group B	3	0	0	0	3
Chlorella group	22	0	0	0	22
Raphidonema group	141,750	160,090	39,181	112,163	453,184
Trebouxia group A	52	0	0	0	52
Trebouxia group B	187	6	8	26	227
Chloroidium group A	11	0	0	0	11
Chloroidium group B	40	0	0	0	40
Chloroidium group C	0	0	0	8	8
Pseudostichococcus group	4	0	0	0	4
Asterochloris group	0	136	0	4	140
Chloromonadinia-snow group A	428	582	6	5,699	6,715
Chloromonadinia-snow group B	778	0	908	2,061	3,747
Chloromonadinia-snow group C	2,535	0	0	0	2,535
Chloromonadinia-snow group D	0	19	0	294	313
Chloromonadinia-snow group E	0	0	0	396	396
Chloromonadinia-snow group F	0	0	0	1,482	1,482
Chloromonadinia-snow group G	0	6	0	167,739	167,745
Total	440,021	1,210,037	267,018	981,533	2,898,609

Supplementary Table 4: Proportion of Illumina read sequences for each taxonomy group in the various regions

	Proportion of each group in total	Region			
		Antarctica	Svalbard	Greenland	Alaska
‘*Chlamydomonas*’-snow group A	6.3	30.9	3.0	2.0	0.4
‘*Chlamydomonas*’-snow group B	71.7	35.5	83.7	83.0	70.0
unnamed group	0.001	0.004	0	0	0
Stephanosphaerinia group	0.01	0.09	0	0	0
Moewusinia group A	0.1	0.4	0.004	0	0
Moewusinia group B	0.0001	0.001	0	0	0
Chlorella group	0.001	0.005	0	0	0
Raphidonema group	15.6	32.2	13.2	14.7	11.4
Trebouxia group A	0.002	0.01	0	0	0
Trebouxia group B	0.008	0.04	0.0005	0.003	0.003
Chloroidium group A	0.0004	0.002	0	0	0
Chloroidium group B	0.001	0.01	0	0	0
Chloroidium group C	0.0003	0	0	0	0.001
Pseudostichococcus group	0.0001	0.001	0	0	0
Asterochloris group	0.005	0	0.01	0	0.0004
Chloromonadinia-snow group A	0.2	0.10	0.05	0.002	0.6
Chloromonadinia-snow group B	0.1	0.2	0	0.3	0.2
Chloromonadinia-snow group C	0.1	0.6	0	0	0
Chloromonadinia-snow group D	0.01	0	0.002	0	0.03
Chloromonadinia-snow group E	0.01	0	0	0	0.04
Chloromonadinia-snow group F	0.1	0	0	0	0.2
Chloromonadinia-snow group G	5.8	0	0.0005	0	17.1
Total	100	100	100	100	100

Supplementary Table 5: PERMANOVA analysis of Illumina ITS2 sequences based on 98% OTU

	Antarctica	Svalbard	Alaska	Greenland
Antarctica				
Svalbard	0.0001*			
Alaska	0.0016*	0.0074*		
Greenland	0.0682	0.2381	0.3333	

* statistically significant after Bonferroni's correction at $P < 0.01$.

Supplementary Table 6: PERMANOVA analysis of Illumina ITS2 sequences based on unique sequences

	Antarctica	Svalbard	Alaska	Greenland
Antarctica				
Svalbard	0.0001*			
Alaska	0.0006*	0.1425		
Greenland	0.0226	0.381	0.1333	

* statistically significant after Bonferroni's correction at $P < 0.01$.

Supplementary Table 7: Mantel test of Illumina ITS2 sequences of algae in red snow samples from the polar regions

	Both Polars	Within Arctic region	Within Antarctic region
Unique sequences	0.0001 *	0.0726	0.0067*
98% OTUs	0.0002*	0.0333	0.366

* statistically significant after Bonferroni's correction at $P < 0.01$.

Supplementary Table 8: Classification of distribution category for this study

Category	Detecting region
Endemic	Antarctica
	Greenlang
	Svalbard
	Alaska
Multiple area	Antarctica Svalbard
	Antarctica - Greenland
	Antarctica - Alaska
	Svalbard - Antarctica
	Svalbard - Greenland
	Svalbard - Alaska
	Greenland - Antarctica
	Greenland - Svalbard
	Greenland - Alaska
	Alaska - Antarctica
	Alaska - Svalbard
	Alaska - Greenland
	Antarctica - Svalbard - Greenland
	Antarctica - Svalbard - Alaska
	Svalbard - Greenland - Alaska
Arctic group	Svalbard - Greenland - Alaska
Cosmopolitan	Antarctica - Svalbard - Greenland - Alaska

Supplementary Table 9: Numbers of endemic distribution sequences for each group of Illumina read sequences

	Endemic				Multiple area	Total
	Antarctica	Svalbard	Alaska	Greenland		
Chlamydomonas-snow group A	136,035	9,388	4,042	5,200	27,750	182,415
Chlamydomonas-snow group B	37,533	57,434	138,819	3,057	1,840,680	2,077,523
unnamed group	16	0	0	0	0	16
Stephanosphaerinia group	386	0	0	0	0	386
Moewusinia group A	1,598	47	0	0	0	1,645
Moewusinia group B	3	0	0	0	0	3
Chlorella group	22	0	0	0	0	22
Raphidonema group	10,6101	16,483	21,825	4,241	304,534	453,184
Trebouxia group A	52	0	0	0	0	52
Trebouxia group B	111	3	0	8	105	227
Chloroidium group A	11	0	0	0	0	11
Chloroidium group B	40	0	0	0	0	40
Chloroidium group C	0	0	8	0	0	8
Pseudostichococcus group	4	0	0	0	0	4
Asterochloris group	0	21	0	0	119	140
Chloromonadinia-snow group A	428	147	2,847	6	3,287	6,715
Chloromonadinia-snow group B	778	0	2061	908	0	3,747
Chloromonadinia-snow group C	2,535	0	0	0	0	2,535
Chloromonadinia-snow group D	0	18	202	0	93	313
Chloromonadinia-snow group E	0	0	396	0	0	396
Chloromonadinia-snow group F	0	0	1,482	0	0	1482
Chloromonadinia-snow group G	0	0	65,265	0	102,480	167,745
Total	285,653	83,541	236,947	13,420	2,279,048	2,898,609

Supplementary Table 10: Proportion of endemic distribution sequences for each group of Illumina read sequences

	Endemic				Multiple area	Total
	Antarctica	Svalbard	Alaska	Greenland		
‘*Chlamydomonas*’-snow group A	74.6	5.1	2.2	2.9	15.2	100
‘*Chlamydomonas*’-snow group B	1.8	2.8	6.7	0.1	88.6	100
unnamed group	100	0	0	0	0	100
Stephanosphaerinia group	100	0	0	0	0	100
Moewusinia group A	97.1	2.9	0	0	0	100
Moewusinia group B	100	0	0	0	0	100
Chlorella group	100	0	0	0	0	100
Raphidonema group	23.4	3.6	4.8	0.9	67.2	100
Trebouxia group A	100	0	0	0	0	100
Trebouxia group B	48.9	1.3	0.0	3.5	46.3	100
Chloroidium group A	100	0	0	0	0	100
Chloroidium group B	100	0	0	0	0	100
Chloroidium group C	0	0	100	0	0	100
Pseudostichococcus group	100	0	0	0	0	100
Asterochloris group	0	15.0	0	0	85.0	100
Chloromonadinia-snow group A	6.4	2.2	42.4	0.1	49.0	100
Chloromonadinia-snow group B	20.8	0	55.0	24.2	0	100
Chloromonadinia-snow group C	100	0	0	0	0	100
Chloromonadinia-snow group D	0	5.8	64.5	0	29.7	100
Chloromonadinia-snow group E	0	0	100	0	0	100
Chloromonadinia-snow group F	0	0	100	0	0	100
Chloromonadinia-snow group G	0	0	38.9	0	61.1	100

Supplementary Table 11: Distribution of the whole-Arctic region group based on Illumina unique ITS2 sequences

	Unique sequences	Unique sequences in group (%)	Unique sequences in total (%)	Sequencing reads	Sequencing reads in group (%)	Sequencing reads in total (%)
‘*Chlamydomonas*’-snow group A	5	0.1	0.01	21,852	12.0	0.8
‘*Chlamydomonas*’-snow group B	965	2.6	1.5	528,992	25.5	18.2
Raphidonema group	255	1.6	0.4	4,842	1.1	0.2
Total	1,225		1.9	555,686		19.2

Supplementary Table 12: Cosmopolitan distribution based on Illumina unique ITS2 sequences

	Unique sequences	Unique sequences in group (%)	Unique sequences in total (%)	Sequencing reads	Sequencing reads in group (%)	Sequencing reads in total (%)
‘*Chlamydomonas*’-snow group B	62	0.17	0.10	842,052	40.5	29.1
Raphidonema group	850	5.48	1.33	240,260	53.0	8.3
Total	912	-	1.42	1,082,312	-	37.3
